# *LINC02470* impairs natural killer cell cytotoxicity by epigenetically targeting the natural cytotoxicity triggering receptor 1

**DOI:** 10.3389/fimmu.2026.1627089

**Published:** 2026-04-16

**Authors:** Xiumin Shi, Lei Zhou, Min Li, Xue Wen, Yongchong Chen, Chao Niu, Qiliang Yin, Haofan Jin, Andrew R. Hoffman, Jiuwei Cui, Sujun Gao, Ji-Fan Hu

**Affiliations:** 1 Department of Hematology, First Hospital, Jilin University, Changchun, Jilin, China; 2 Cancer Center, First Hospital, Jilin University, Changchun, Jilin, China; 3 Department of Cadre Ward, First Hospital, Jilin University, Changchun, Jilin, China; 4 Department of Medicine, Stanford University Medical School, Stanford, CA, United States

**Keywords:** NK cells, cytotoxicity, LINC02470, NCR1, intrachromosomal interaction, histone modification, enhancer RNA

## Abstract

**Introduction:**

Natural killer cells (NK cells) play a critical role in the surveillance of tumor immunity. However, NK cell-based immunotherapy, including autologous and allogeneic NK cell reinfusion, has not brought significant clinical benefits to patients.

**Methods:**

To identify factors that control the intrinsic cytotoxicity of NK cells, we utilized the histone deacetylase inhibitor valproic acid (VPA) to develop a NK cell cytotoxicity suppression model. With RNA-seq and functional assays, we identified a lncRNA, *LINC02470*, as a negative regulatory factor of NK cell-mediated cytotoxicity. *LINC02470* was significantly upregulated in VPA-treated NK cells and was negatively associated with the cytotoxicity of NK cells. Knockdown of *LINC02470* enhanced, and overexpression of *LINC02470* suppressed antitumor activity in NK-92MI cells and human primary NK cells. Then reverse transcription-associated capture sequencing (RAT-seq) and mechanistic studies were explored to find the target and mechanisms of *LINC02470*.

**Results:**

By RAT-seq we found that *LINC02470* functioned by targeting the Natural cytotoxicity triggering receptor 1 (*NCR1*) gene, which encodes the activating receptor NKp46 involved in the natural cytotoxicity. Mechanistic studies revealed that *LINC02470* interacted with the regulatory elements of *NCR1* and blocked the formation of an intrachromosomal interaction that is required for optimal expression of *NCR1*. In addition, *LINC02470* inhibited the synthesis of *NCR1* enhancer RNA. Through these dual mechanisms, *LINC02470* induced a suppressive epigenotype in the *NCR1* promoter and suppressed the expression of the *NCR1* gene.

**Discussion:**

The *LINC02470-NCR1* axis identified in this study may serve as a novel target to improve therapeutic intervention of NK cells in tumor immunotherapy.

## Introduction

Liver cancer is the sixth most common malignancy, and hepatocellular carcinoma (HCC) (80% of primary liver cancers) is one of the three leading causes of cancer-related deaths worldwide (1). Most patients are diagnosed with advanced disease when surgical and locoregional treatments are not feasible or effective. Multikinase inhibitors, like sorafenib, are expensive and can prolong the overall survival for only 3 months. Similarly, regorafenib, cabozantinib, and ramucirumab only improve overall survival for 2.8 months, 2.2 months, 1.2 months, respectively, when compared with placebo in sorafenib resistant patients (2). The efficacy of immunotherapy in HCC remains limited. HCC is the only major cancer for which death rates have not improved over the last 10 years (3), and new therapeutic breakthroughs are urgently needed.

Natural killer cells (NK cells) are important components of innate immunity. They play an important role in the surveillance of tumor immunity and are closely related to the occurrence, development and prognosis of a variety of human tumors (4–6). Studies have shown that the number and function of NK cells are decreased in peripheral blood of patients with HCC (7–10), and are significantly correlated with the survival and prognosis of HCC patients (11, 12). However, NK cell-based immunotherapy, such as autologous and allogeneic NK cell reinfusion, cytokine therapy, etc. has not brought significant clinical benefits to patients (13).

Long non-coding RNAs (lncRNAs) are a class of RNA molecules longer than 200 nucleotides that are not translated into peptides. LncRNAs can regulate the expression of genes by forming complex structures with DNA, by regulating other epigenetic mechanisms, and by mediating the formation of circular structures in chromosomes. It remains unclear how lncRNAs affect gene expression in human NK cells, and little has been reported about the role of lncRNAs in NK cells. Wright et al. (14) reported that overexpression of *KIR* antisense lncRNA caused decreased expression of the gene encoding KIR protein. *Lnc-CD56* may have a positive regulatory effect on CD56 in human NK cells (15). LncRNA *NCAL1* potentiates natural killer cell cytotoxicity through the Gab2-PI3K-AKT pathway (16).

We previously found that the histone deacetylase inhibitor valproic acid (VPA) suppressed NK cell cytotoxicity (17). However, the specific mechanism underlying the role of VPA in NK cell cytotoxicity remains unknown. To identify factors that may regulate NK cell activity, we performed next-generation sequencing (NGS) on activated human NK cells treated with VPA. LncRNA *LINC02470* is located within a functional domain on chromosome 12 that contains C-type lectin domain family members and the killer cell lectin-like receptor family members involved in natural killer cell-mediated cytotoxicity. *LINC02470* was identified as a potential negative factor that regulates NK cell-mediated cytotoxicity. The mechanisms underlying the suppression of NK cell cytotoxicity by *LINC02470* was also investigated.

## Results

### RNA-seq identifies LINC02470 as a NK cytotoxicity-associated lncRNA

**Figure 1:**
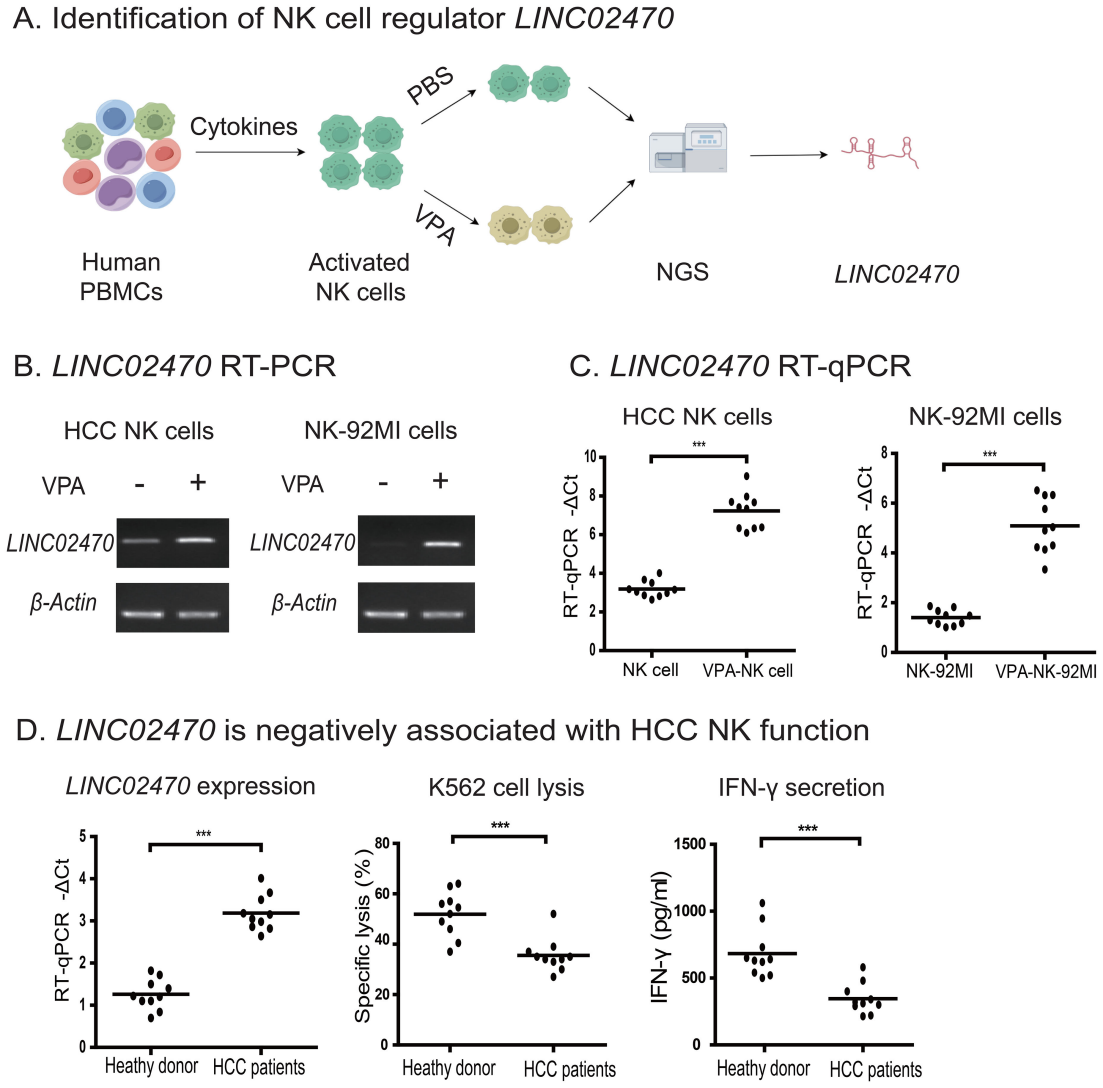
Identification of lncRNA *LINC02470* as a negative regulator of NK cytotoxicity. **(A)** Identification of NK cell regulator *LINC02470*. **(B)** *LINC02470* expression by RT-PCR. VPA-untreated and treated HCC NK cells, VPA-untreated and treated NK-92MI cells were collected for RT-PCR. A representative agarose gel electrophoresis is shown. **(C)** Quantitation of *LINC02470* expression by RT-qPCR in NK cells from HCC patients (n=10) and NK-92MI cells untreated and treated by VPA. ***p<0.001. **(D)** Negative association between *LINC02470* expression and NK cytotoxicity in NK cells from healthy donors (n=10) and HCC patients (n=10). ***p<0.001.

In a previous study, we used histone deacetylase inhibitor VPA to establish a NK cell suppression model to screen regulatory factors involved in NK cell function (17). VPA treatment significantly decreased NK cell cytotoxicity in lung cancer patients. To identify molecular factors that regulate NK cell function, we performed RNA-Seq on VPA-treated cells (**Supplementary Figures S1**–**S3**). We identified many lncRNAs that were associated with NK cytotoxicity, and they were designated as NK cytotoxicity-associated long noncoding RNAs (NKCLRs). Functional assays were then conducted for these identified lncRNAs. Notably, a 979 bp lncRNA *LINC02470* was located in a unique NK cell functional domain on chromosome 12 (**Supplementary Figure S4**) that contains the C-type lectin domain family member cluster (*CLEC2A, CLEC12A, CLEC2B, CLEC1B, CLEC12B, CLEC9A, CLEC1A, CLEC7A, CLEC2D*) involved in natural killer cell-mediated cytotoxicity and the killer cell lectin-like receptor family members (*KLRF2, KLRF1, KLRB1, KLRD1, KLRC4, KLRC2, KLRC1*) that function upstream of or within a region associated with natural killer cell activation and positive regulation of natural killer cell mediated cytotoxicity. Therefore, *LINC02470* was selected for further mechanistic studies (**Figure 1A**).

### LINC02470 is negatively associated with the antitumor activity of NK cells

We first examined the association between *LINC02470* expression and the function of NK cells. NK cells were isolated from peripheral blood of HCC patients by magnetic beads and were treated with VPA as previously described (17). The NK-92MI cell line was also treated in parallel with VPA as a control. As expected, VPA treatment reduced the activity of tumor cell lysis in both HCC NK cells and NK-92MI cells. Similar results were also observed when NK function was assessed by IFN-γ secretion (**Supplementary Figure S5**). Notably, *LINC02470* was significantly upregulated in these VPA-treated cells, and there was a negative association between *LINC02470* abundance and the antitumor activity of NK cells (**Figures 1B, C**).

We also compared *LINC02470* expression with NK functions in NK cells collected from a cohort of HCC patients and healthy subjects. NK cells from HCC patients expressed significantly more *LINC02470* than in healthy donors, in parallel with lower NK function (**Figure 1D**). These data suggest that *LINC02470* expression is negatively associated with the antitumor activity of NK cells.

### LINC02470 knockdown enhances but LINC02470 overexpression suppresses the cytotoxicity of NK-92MI cells

**Figure 2:**
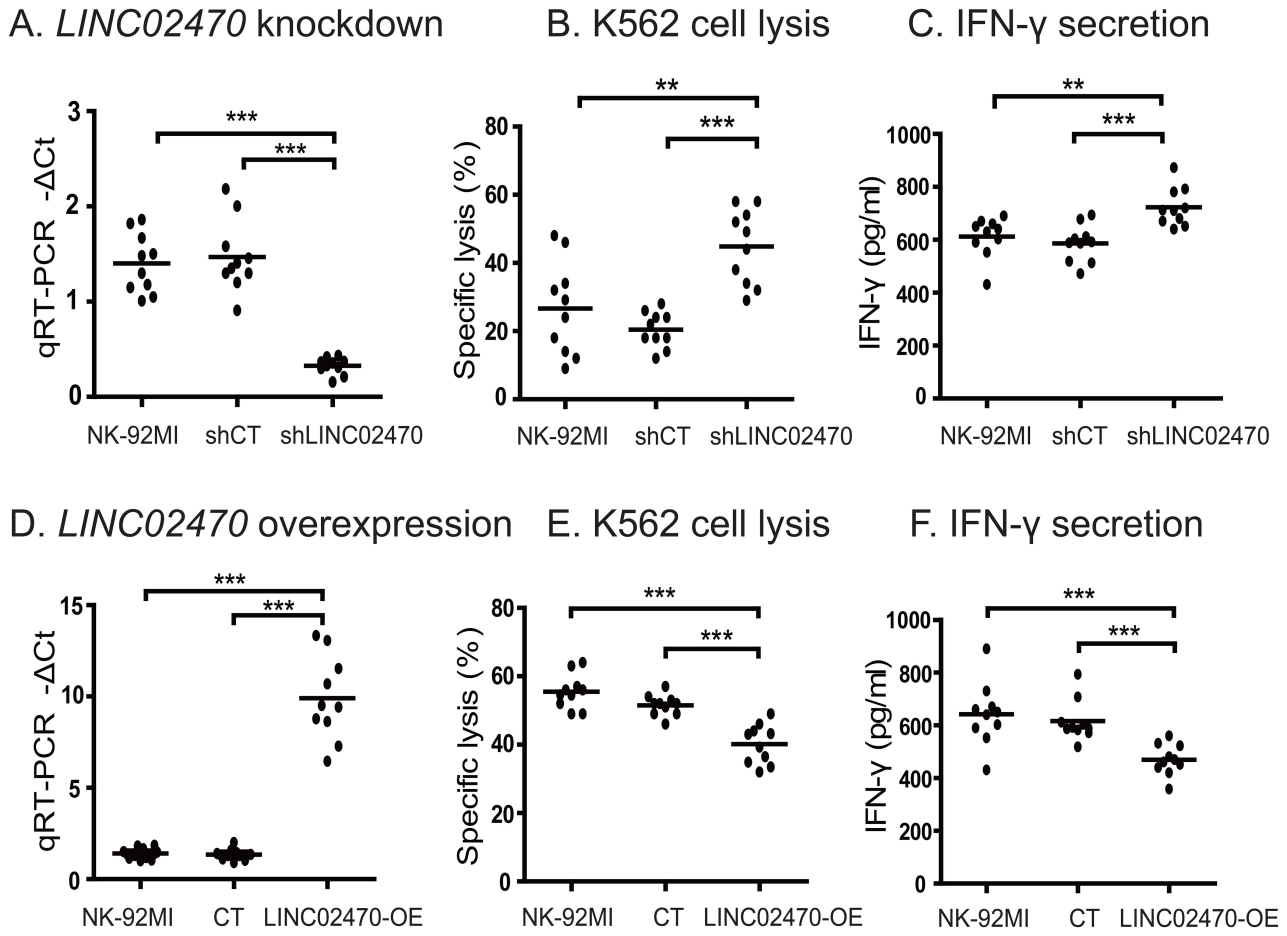
*LINC02470* knockdown increased **(A–C)**, and *LINC02470* overexpression suppressed **(D–F)** the cytotoxicity of NK-92MI cells. shCT: NK-92MI transfected with random control shRNA vector; shLINC02470: NK-92MI transfected with shLINC02470 vector. CT: NK-92MI transfected with blank control plasmid. *LINC02470*-OE: NK-92MI transfected with *LINC02470* overexpression plasmid. Data represent ten independent experiments. ***p<0.001. **p<0.01.

We then examined the function of *LINC02470* in NK cells by shRNA knockdown. The shLINC02470 plasmid was constructed and lentiviruses were packaged in 293SF-PacLV packing cells. NK-92MI cells were transduced with shLINC02470 lentiviruses (**Supplementary Figure S6**). After shRNA knockdown, the relative expression of *LINC02470* was detected by qPCR. The antitumor activity of NK cells was evaluated by specific lysis using the calcein-AM assay and IFN-γ secretion by ELISA. We showed that knockdown of *LINC02470* enhanced the antitumor activity in NK-92MI cells as shown by both tumor cell specific lysis and IFN-γ secretion assays (**Figures 2A–C**).

We also examined the role of *LINC02470* overexpression in NK-92MI cells. *LINC02470* lncRNA was cloned in a lentiviral vector and packaged in 293T cells. Lentiviruses were transduced into NK-92MI cells. We showed that overexpression of *LINC02470* suppressed the tumor cell specific lysis and IFN-γ secretion in NK-92MI (**Figures 2D–F**).

**Figure 3:**
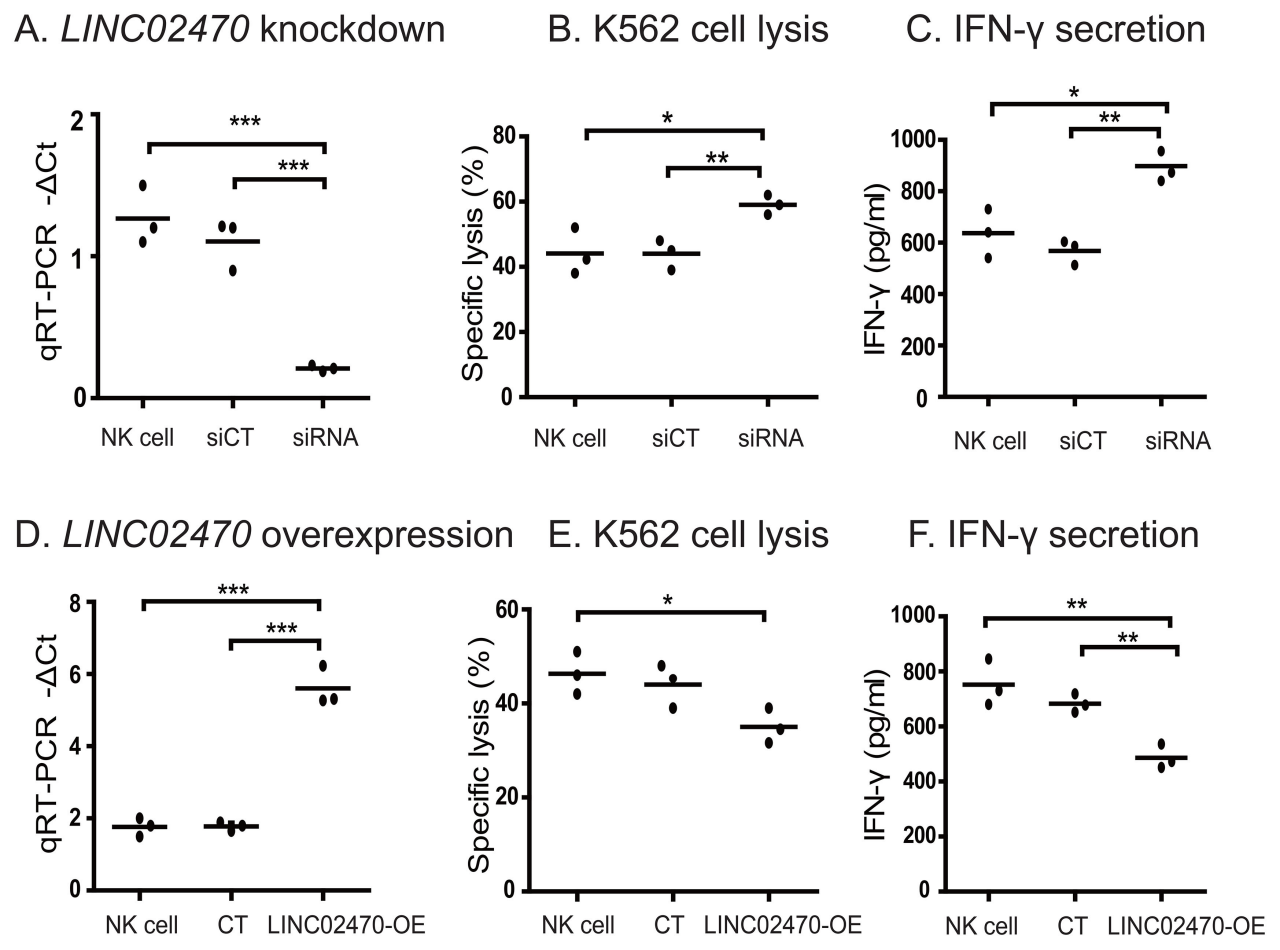
The role of *LINC02470* in primary human NK cells. **(A–C)**. *LINC02470* siRNA knockdown in primary human NK cells. siCT, primary human NK cells transfected with random siRNA; siRNA: primary human NK cells transfected with siRNA for *LINC02470*. Primary NK cells were from one healthy donor. Data represent three independent experiments. **(D–F)**. Overexpression of *LINC02470* in primary human NK cells. CT, primary human NK cells transfected with blank control plasmid. *LINC02470*-OE: primary human NK cells transfected with *LINC02470* overexpression plasmid. ***p<0.001. **p<0.01. *p<0.05. Primary NK cells were from one healthy donor. Data represent three independent experiments.

In addition to NK-92MI cells, we also examined the role of *LINC02470* in human primary NK cells isolated from healthy donors. A panel of *LINC02470* siRNAs were transfected into human primary NK cells. Again, we showed that *LINC02470* knockdown significantly enhanced the activity of K562 specific lysis and IFN-γ secretion (**Figures 3A–C**). On the other hand, overexpression of *LINC02470* suppressed the cytotoxicity of human primary NK cells (**Figures 3D–F**). These data were comparable to that obtained in NK-92MI cells.

### Subcellular location of LINC02470 in NK-92MI cells

**Figure 4:**
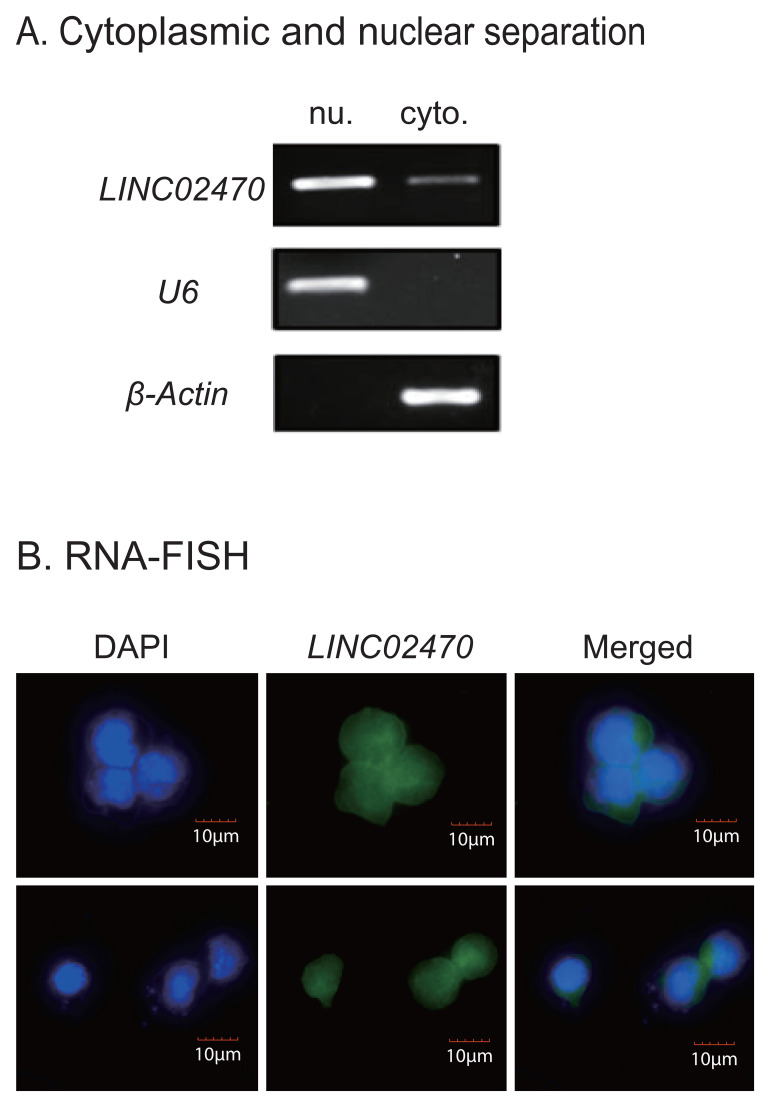
Location of *LINC02470* in NK-92MI cells. **(A)** *LINC02470* location assays by cytoplasmic and nuclear separation and PCR. U6: nuclear control. β-Actin: cytoplasm control. **(B)** Confirmation of *LINC02470* location by RNA-FISH. DAPI: nuclear control. Both results showed that *LINC02470* was primarily located in the nucleus.

The functions of lncRNAs are closely associated with their subcellular localization. We isolated cytoplasmic and nuclear RNA and used RT-qPCR to quantitate the subcellular distribution of *LINC02470* in NK cells. We found that *LINC02470* was located dominantly in the nucleus (**Figure 4A**). RNA fluorescence *in situ* hybridization (RNA-FISH) also confirmed the nuclear location of *LINC02470* (**Figure 4B**). These data suggest that *LINC02470* might affect the antitumor function of NK-92MI cells at the transcription level in the nucleus.

### LINC02470 decreases NK-92MI cell cytotoxicity by downregulating NCR1

**Figure 5:**
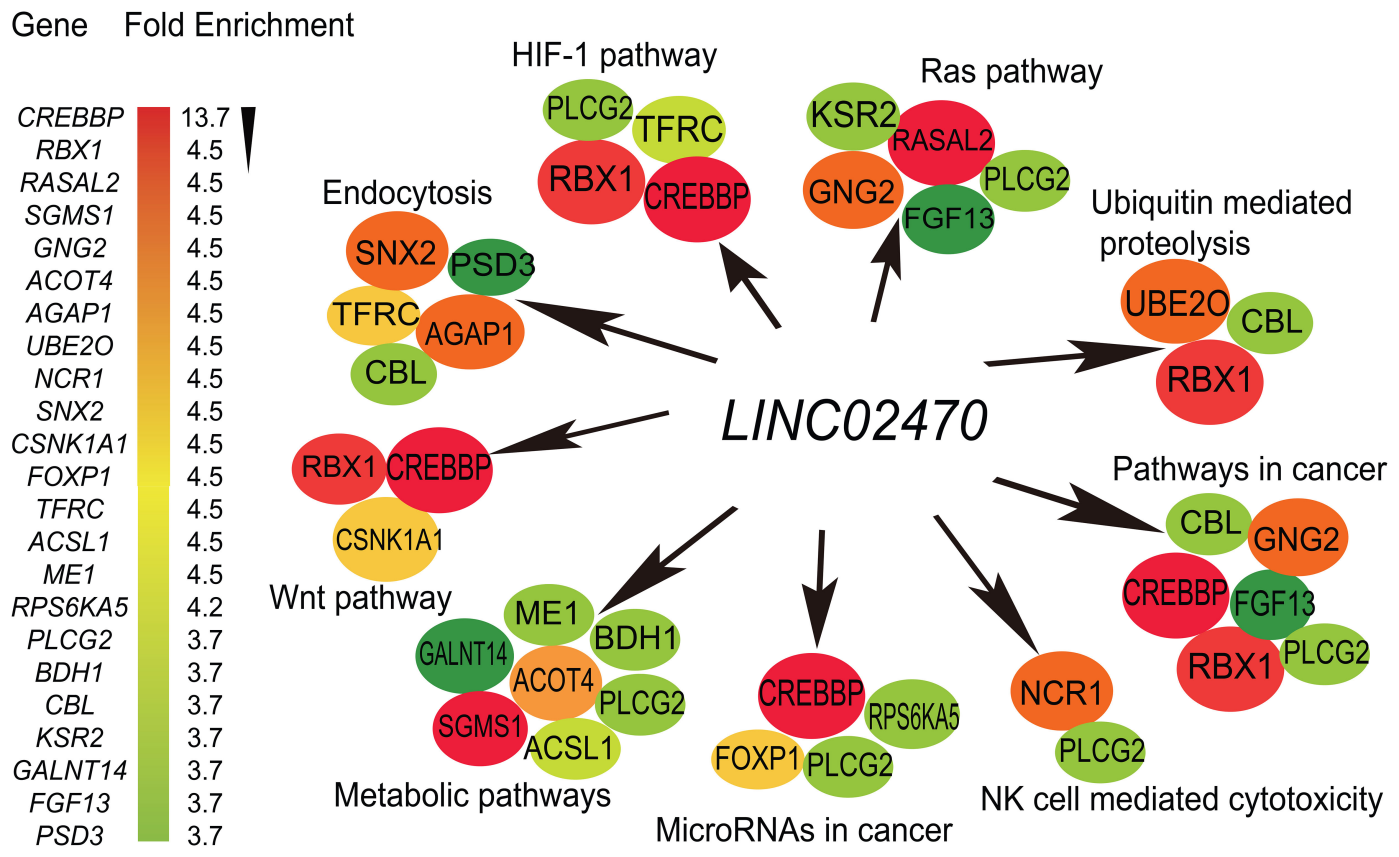
The *LINC02470* RAT-seq interactome. The *LINC02470* interactome was drawn based on the enrichment fold of the top RAT-seq pathway target genes. *LINC02470* binds to many pathway genes that are involved in NK cell mediated cytotoxicity, metabolic pathways, pathways in cancer, endocytosis, etc.

To explore the underlying mechanisms by which *LINC02470* regulates NK-92MI cell cytotoxicity, we used a reverse transcription-associated capture sequencing (RAT-seq) assay (18, 19) to profile the genome-wide targets of *LINC02470*. Bioinformatics analysis revealed that *LINC02470* interacted with 195 target genes. Gene Ontology enrichment pathway and RAT-seq interactome map analyses showed that *LINC02470* interacted with genes involved in the NK cell cytotoxicity, endocytosis, ubiquitination, and HIF-1 pathway (**Figures 5**; **Supplementary Figure S7**).

**Figure 6:**
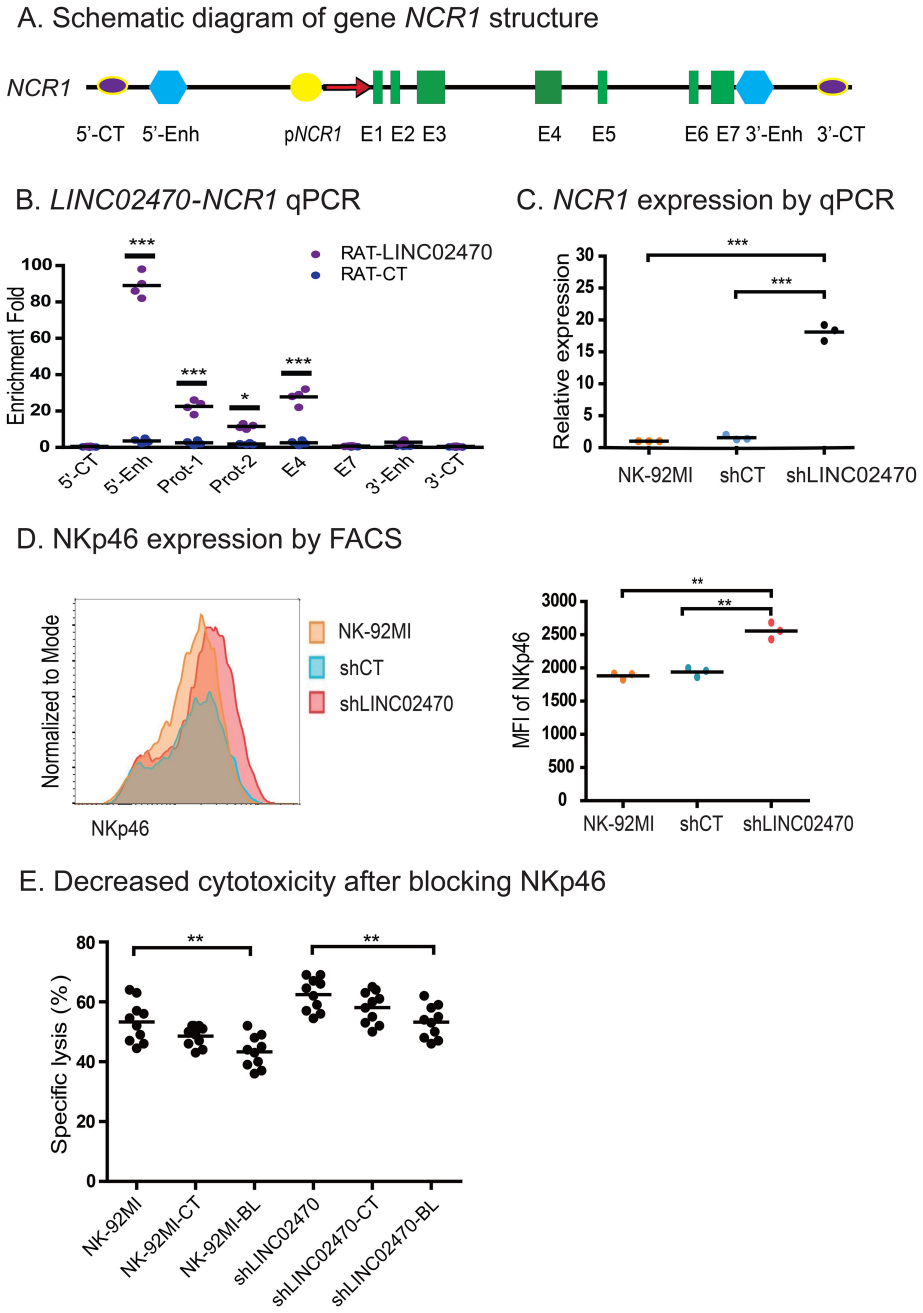
*LINC02470* suppressed the expression of *NCR1*. **(A)** Schematic diagram of gene *NCR1* structure. 5’-Enh, 3’-Enh: the *NCR1* 5’- and 3’-enhancers; p*NCR1*: *NCR1* promoter; E1-E7: *NCR1* exons. 5’-CT, 3’-CT: the RAT control sites. **(B)** Primers were designed in different regions of *NCR1* gene, and the RAT pulldown complex was used to map the *LINC02470* binding by qPCR. Note the enrichment of the *LINC02470* binding signals in the 5’-enhancer and promoter region (5’-Enh, Prot-1 and Prot-2) (***P < 0.001, *P < 0.05 as compared with the RAT control). Data represent four independent experiments. **(C)** The relative expression of *NCR1* was detected by qPCR in NK-92MI, shCT and shLINC02470 cells, respectively. The results showed that *NCR1* expression significantly increased after *LINC02470* knockdown. Experiments were performed in triplicate. ***P < 0.001. **(D)** Representative flow cytometry analysis of NKp46 expression in NK-92MI and shLINC02470 cells. Both percentage and mean fluorescence intensity (MFI) of NKp46 expression increased after knocking down *LINC02470*. Three independent experiments were performed. **P < 0.01. **(E)** The role of NKp46 in NK cell cytotoxicity. Both NK-92MI cells and shLINC02470 cells were pre-incubated with anti-NKp46 antibody and were then tested for killing of K652 cells. Data were obtained from ten independent experiments. NK-92MI-CT: NK-92MI cells pre-incubated with an isotype control IgG; NK-92MI-BL: NK-92MI cells pre-incubated with anti-NKp46 antibody; shLINC02470: NK-92MI transfected with shLINC02470 vector; shLINC02470-CT: shLINC02470 cells pre-incubated with an isotype control IgG; shLINC02470-BL: shLINC02470 cells pre-incubated with anti-NKp46 antibody. **P < 0.01.

Of all the *LINC02470* target genes, we focused on the Natural cytotoxicity triggering receptor 1 (*NCR1*) gene, which encodes NKp46 and is closely related to function of NK cells. We used RAT-qPCR to map the binding sites of *LINC02470* in different regions of the *NCR1* gene locus. The RAT pulldown complex was quantitated for the abundance of *LINC02470* binding signals. *LINC02470* binding signals were primarily located at the 5’-enhancer and promoter regions of the *NCR1* gene (**Figures 6A, B**), suggesting that *LINC02470* may function through these regulatory elements. No RAT signals were detected at other regions, including the 5’- and the 3’- control sites (5’-CT, 3’-CT) and the 3’- enhancer. No such interaction signals were detected in the RAT random control library products.

We then examined the role of *LINC02470* in the regulation of *NCR1* in NK cells. *LINC02470* was knocked down by lentiviruses carrying *LINC02470* shRNA. We found that knockdown of *LINC02470* significantly enhanced the expression of *NCR1* in NK cells (**Figure 6C**). The abundance of the *NCR1*-encoded protein NKp46 was also confirmed by flow cytometry. As expected, both the percentage and the mean fluorescence intensity (MFI) of NKp46 expression increased after *LINC02470* knockdown (**Figure 6D**). Collectively, these data suggest that *LINC02470* binds to the 5’-enhancer and promoter elements, where it downregulates *NCR1* in NK cells.

It should be noted that *LINC02470* shRNA knockdown caused an approximately 15-fold increase in *NCR1* gene transcription (**Figure 6C**). However, this increase in *NCR1* mRNA did not fully translate into the level of NKp46 protein on the cell surface as measured by FACS (**Figure 6D**). It seems that a posttranscriptional regulation mechanism of the *NCR1* gene may be involved in this discrepancy.

Additionally, we also examined the contribution of NKp46 to K-562 cell killing by blocking its function using a NKp46 blocking antibody. As a pivotal NK activating receptor, NKp46 participates in recognition and activation of NK cells against pathogens, tumor cells, and virally infected cells. As expected, blocking NKp46 with anti-NKp46 antibody reduced the cytotoxicity of NK-92MI cells (**Figure 6E**). *LINC02470* reduced the cell cytotoxicity of NK-92MI by downregulating *NCR1*, the encoding gene of NKp46. We found that pre-incubation with anti-NKp46 antibody abolished the therapeutic effect of shLINC02470 in NK-92MI cells (**Figure 6E**). These data confirm the critical role of NKp46 in the function of NK cells.

### LINC02470 orchestrates chromatin looping in the NCR1 locus

**Figure 7:**
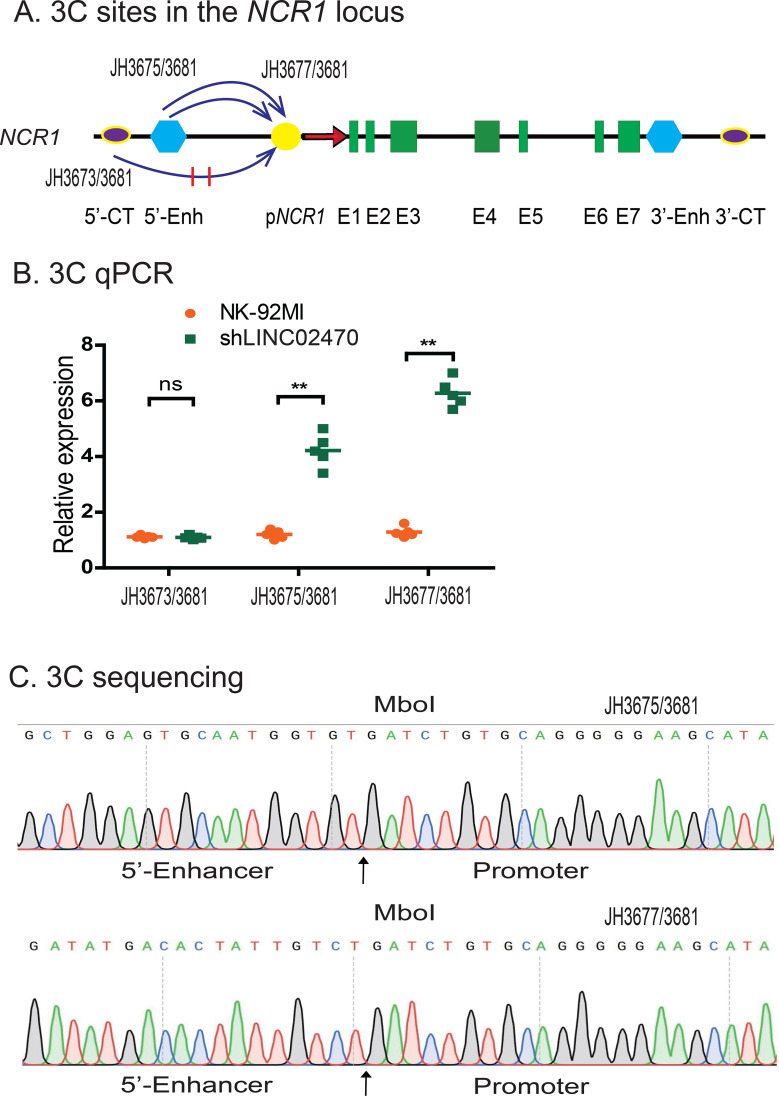
*LINC02470* damaged the maintenance of intrachromosomal looping in the *NCR1* locus. **(A)** The PCR sites in the *NCR1* locus. 5’-Enh: 5’-enhancer; p*NCR1*: *NCR1* promoter; E1-E7: Exons; 3’-Enh: 3’-Enhancer. 5’-CT, 3’-CT: the RAT control sites. Arrows: intrachromosomal interactions. **(B)** Intrachromosomal interactions were quantitated by 3C (chromatin conformation capture) qPCR. Knockdown of *LINC02470* raised intrachromosomal interaction loops. Data were obtained from five independent experiments. For comparison, the relative 3C interaction was calculated by setting the 5’- and 3’- controls as 1 (**P < 0.01 as compared with the NK-92MI controls). **(C)** Sequencing of the *NCR1* intrachromosomal loop products. Arrows: the 3C ligation product containing the MboI site that is flanked by the promoter and the enhancer sequences.

To determine the specific mechanism by which *LINC02470* negatively regulates *NCR1*, we used a chromosome conformation capture (3C) approach to examine intrachromosomal looping, an important epigenetic mechanism involved in gene regulation. NK-92MI and shLINC02470 cells were crosslinked and lysed, followed by MboI digestion and T4 DNA ligation. After proteinase K treatment, DNA was purified for 3C qPCR using primers from different regions of *NCR1*, including the 5’ -enhancer and the promoter (**Figure 7A**). Knockdown of *LINC02470* enhanced the intrachromosomal loop between the 5’ -enhancer and the promoter in shLINC02470 cells (**Figure 7B**). No intrachromosomal looping was detected between the control sites (5’-CT) and the promoter.

We validated these intrachromosomal loops by DNA sequencing of 3C PCR products. As expected, we observed that the MboI ligation site was flanked by the sequences of the *NCR1* 5’ -enhancer and promoter (**Figure 7C**). These data suggest that *LINC02470* binds to the *NCR1* regulatory elements and blocks the formation and/or maintenance of these specific intrachromosomal interactions required for optimal expression of *NCR1*. Knockdown of *LINC02470* enhanced the three-dimensional chromatin structures that activate the *NCR1* gene to maintain the NK cell activity.

### LINC02470 inhibits the NCR1 enhancer RNA pathway

Enhancers are DNA elements that act over a distance to positively regulate expression of target genes in a spatial and temporal fashion, primarily through the formation of three-dimensional chromatin structures. Active enhancers, especially super enhancers, possess transcription potential, generating bidirectional non-coding transcripts, defined as enhancer RNAs (eRNAs), to regulate the gene activity (20).

**Figure 8:**
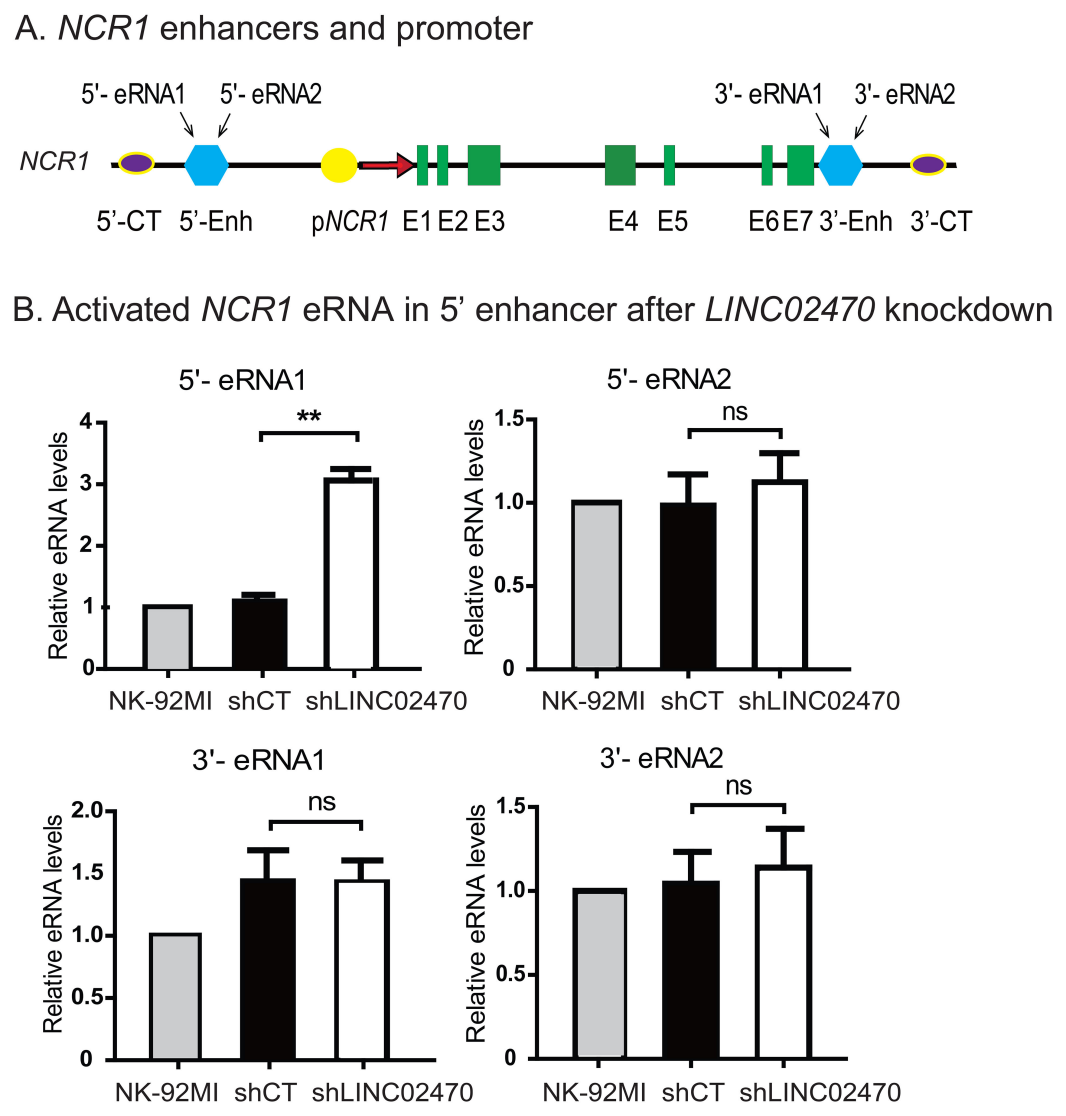
*LINC02470* inhibits the *NCR1* enhancer RNA pathway. **(A)** Location of *NCR1* enhancer RNAs. **(B)** *LINC02470* knockdown activates *NCR1* eRNA in 5’ enhancer. Expression of *NCR1* eRNAs in NK-92MI, shCT and shLINC02470 cells was measured by RT-qPCR. shCT: NK-92MI transfected with the random shRNA vector control; shLINC02470: NK-92MI transfected with shLINC02470. Gene expression was normalized to β-Actin control. All experiments were performed in triplicate and statistically significant differences by Student’s t test. **P < 0.01.

To determine if *LINC02470* might regulate *NCR1* through eRNA, we collected NK-92MI, shCT and shLINC02470 cells and used qPCR to measure *NCR1* eRNAs that would include the 5’- enhancer and 3’- enhancer (**Figure 8A**). The amount of eRNA1 at the *NCR1* 5’- enhancer sites was significantly upregulated in shLINC02470 cells (**Figure 8B**), suggesting that *LINC02470* may regulate *NCR1* gene activity by inhibiting eRNAs synthesis.

### LINC02470 inhibits NCR1 by enriching histone suppressive markers in the gene promoter

Since both intrachromosomal looping and eRNAs are important epigenetic drivers that induce epigenetic modifications in the gene promoter, we examined if *LINC02470* uses these mechanisms to alter epigenotypes in the *NCR1* gene promoter. First, we examined the status of DNA methylation in the *NCR1* promoter. We collected NK-92MI, shLINC02470 and shCT cells, extracted genomic DNA, and used EZ DNA Methylation-Gold Kit to map DNA methylation in the *NCR1* promoter (**Supplementary Figure S8A**). *LINC02470* knockdown did not affect the status of CpG DNA demethylation in shLINC02470 cells (**Supplementary Figure S8B**), indicating that *LINC02470* regulates *NCR1* through a non-DNA methylation mechanism in NK-92MI cells.

**Figure 9:**
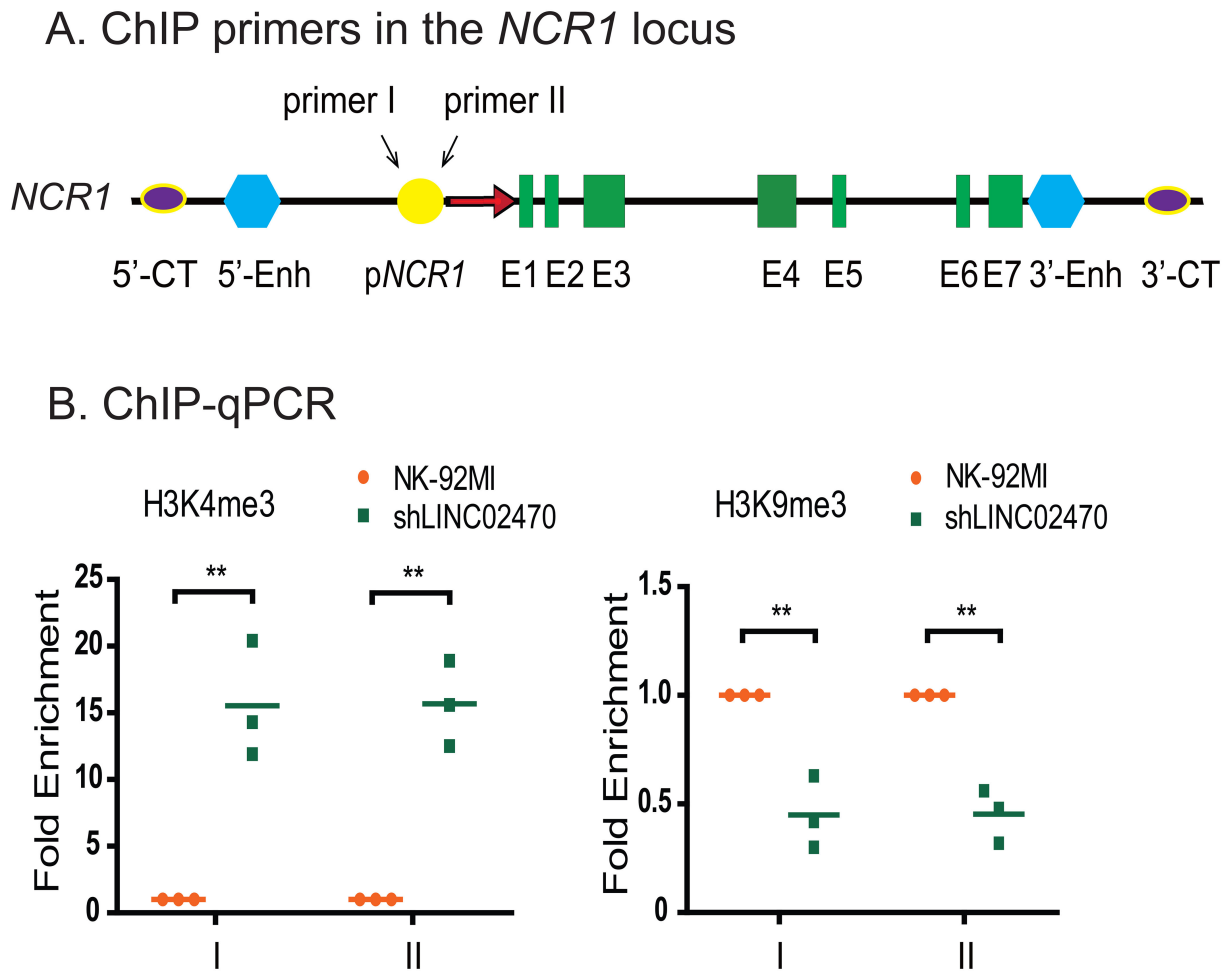
*LINC02470* abolished enrichments of *NCR1* DNA sequences for H3K4me3, but enhanced H3K9me3 in the promoter of *NCR1* gene. Two pairs of primers were designed in the promoter of *NCR1* gene. Enrichments of *NCR1* DNA sequences for H3K4me3, H3K9me3 occupancy in NK-92MI and shLINC02470 cells were measured using ChIP-qPCR. Data were obtained from three independent experiments. **p < 0.01.

We then examined the status of histone modifications histone H3K9me3, H3K4me3, H3K27ac, and H3K9ac using ChIP assays. Two pairs of primers in the promoter of *NCR1* gene were selected for qPCR quantitation. *LINC02470* knockdown enhanced the enrichment of active histone maker H3K4me3, but reduced the suppressive histone marker H3K9me3 level in the *NCR1* promoter (**Figure 9**). However, *LINC02470* had no significant effect on the enrichments of H3K27ac and H3K9ac (**Supplementary Figure S9**). These results further suggest that *LINC02470* inhibits *NCR1* gene expression by modifying the epigenotypes in the *NCR1* promoter.

## Discussion

Little is currently known about the role of lncRNAs in NK cells. One reason is the lack of appropriate experimental models. In our previous study, NK cells were treated with VPA to establish an inhibitory model to detect the function of lncRNA in NK cells. Using RNA-Seq and bioinformatic analysis, we identified *LINC02470*, which impairs the cytotoxicity of NK cells.

LncRNA localization is closely related to its function (21). In this study, *LINC02470* is primarily localized in the nucleus, indicating that it may regulate the function of NK cells at the gene transcriptional level. Through RAT-seq and bioinformatics analysis, we identified *NCR1* (encoding NKp46), a gene that is upregulated by *LINC02470* knockdown in NK-92MI cells. NKp46, a major lysis receptor, is one of the most evolutionarily ancient NK cells activating receptors. The activation of NKp46 resulted in direct killing of target cells (22, 23). Our study showed that *LINC02470* affected the expression of *NCR1* and thus the activity of NK cells. In addition to exogenous ligands, such as viral haemagglutinins and fungal adhesins, ecto-calreticulin (ecto-CRT) has been recently identified as a new endogenous ligand for NKp46 (24). Using qPCR, we found that ecto-CRT was significantly upregulated in K562 cells as compared with that in healthy bone marrows (**Supplementary Figure S10**). Future studies are needed to address if high ecto-CRT expression in K562 cells is related to its sensitivity to NK cytotoxicity.

Chromatin looping between core promoter elements and distal enhancer elements is critical for gene activation (25, 26). The data from this study show that *LINC02470* is enriched in the *NCR1* regulatory elements, including the 5’-enhancer and promoter. Knockdown of *LINC02470* in NK-92MI cells increases this intrachromosomal interaction, indicating that *LINC02470* acts like a chromatin factor that abolishes the intrachromosomal looping between 5’-enhancer and promoter in *NCR1* locus.

Pol II-transcribed enhancer RNAs (eRNAs) represent an important marker of active enhancers (27). Numerous studies (28, 29) have shown a potential role of eRNAs in the process of chromosome loop formation between enhancers and promoters. eRNAs can also recruit transcriptional activators to the promoter of the target gene (30, 31). In this study, we detect the presence of eRNA1 transcribed from the *NCR1* 5’- enhancer. *LINC02470* knockdown significantly upregulates eRNA expression. Thus, *LINC02470* binds to *NCR1* 5’-enhancer and promoter, where it regulates the *NCR1* gene activity by coordinating eRNAs synthesis and intrachromosomal looping.

H3K4me3 is associated with transcriptional activation, and H3K9me3 is associated with transcriptional suppression (32–34). DNA methylation is associated with histone modifications, and the interplay of these epigenetic modifications is crucial to the functioning of the genome by modifying chromatin architecture (35). Using a chromatin immunoprecipitation assay, we found a significant increase in H3K4me3 levels and a decrease in H3K9me3 level at the promoter of the *NCR1* gene in shLINC02470 cells. However, *LINC02470* does not affect DNA methylation in the *NCR1* promoter.

LncRNAs have been shown to be associated with NK cell function under some pathological conditions. For example, in natural killer/T-cell lymphoma (NKTCL), lncRNAs *ZFAS1, TERC, MIR155HG*, and *SNHG5* were all upregulated, and involved in NKTCL pathology (36). In a specific type of NKTLC, called extranodal natural killer/T-cell lymphoma (ENKL), lncRNA *XIST* (X-inactive specific transcript) enhances Bcl-w expression level, and promotes ENKL cell proliferation by sponging miR-497 (37). In viral infections, lncRNA *MALAT1* reduces IFN-γ expression. Treatment with tenofovir disoproxil fumarate (TDF) in pregnant women with HBV infection could contribute to elevated levels of IFN-γ in NK cells by reducing *MALAT1* (38). Upon exposure to IFN-β, exosomal levels of linc-EPHA6–1 which is secreted by ZIKV infected A549 cells are elevated, leading to suppression of NK cell cytotoxicity against Zika Virus by targeting miR-4485-5p that sponges NKp46 (39). In gastrointestinal diseases, lncRNA *SNHG10*, which is an exosomal secreted lncRNA, attenuates the cytotoxicity of NK cells by directly targeting inhibin subunit beta C. NK cell inhibition contributes to increased tumor growth, and therefore is associated with poor prognosis in colorectal cancer (40). LncRNA-GAS5 promotes NK cell activity against gastric cancer by reducing miR-18a (41). Finally, Li et al. reported a cluster of lncRNA (32 of them increased and 35 of them decreased) expression profiles of decidual natural killer cells in patients with early missed abortion through the interaction with mRNAs (42).

LncRNAs can also affect gene expression through miRNA sponging. The sponging effects of lncRNAs on miRNAs have an important effect on modulating the impact of miRNAs on genes and signaling pathways. PI3K-Akt, MAPK, Wnt and TGF-β are the main pathways affected by miRNAs. Based on the importance of NK cells in the pathogenesis of human HCC, identification of the regulatory effects of *LINC02470* on NK cells could pave the way for considering immunotherapeutic options. Some lncRNAs that affect the activity of NK cells have been found to influence survival time of patients with malignancies (40). Since lncRNAs can be measured in body fluids, they can also serve as non-invasive biomarkers. In our study, the decreased NK cell activity in HCC patients was related to higher *LINC02470* expression, indicating that *LINC02470* may serve as a novel HCC marker. It should also be noted that in addition to *LINC02470*, there are other factors that may also contribute to the decreased lysis of K562 cells and IFN-γ secretion in HCC patients. Further studies are needed to explore the role of those factors, including *LINC02470*, in the prognosis of HCC patients.

In conclusion, we propose that *LINC02470* affects the occurrence and development of HCC by regulating the antitumor activity of NK cells. This study may provide the basis for improving the function of NK cells in HCC patients and might suggest new therapeutic options for NK cell anti-tumor therapy.

## Materials and methods

### Cell isolation and culture

Human erythroleukemic K562 cells were purchased from the American Type Culture Collection (ATCC, VA) and routinely cultivated in RPMI-1640 medium plus 10% heat-inactivated fetal bovine serum (FBS) and 100 U/ml streptomycin-penicillin in a humidified atmosphere containing 5% CO_2_.

NK-92MI cells (Procell Life Science & Technology Co., Ltd., Wuhan, China) were cultured in α-minimum essential medium (Gibco, Grand Island, NY, USA) containing 2 mM L-glutamine (Gibco), 1.5 g/L sodium bicarbonate (Sigma-Aldrich, St. Louis, MO, USA), 0.2 mM inositol, 0.1 mM β-mercaptoethanol (Sigma-Aldrich), 0.02 mM folic acid (Sigma-Aldrich), 12.5% horse serum (Gibco), and 12.5% fetal bovine serum (Natocor, Córdoba, Argentina).

The bone marrow from healthy donor was treated with erythrocyte lysis buffer (Abcam, catalog ab204733). After mix and subsequent centrifugation, the resulting cell pellet was collected.

Peripheral blood mononuclear cells (PBMCs) were isolated from healthy donors and hepatocellular carcinoma patients by Ficoll (Lymphoprep) gradient density centrifugation. Primary NK cells were isolated from human PBMCs using a MACSxpress NK Cell Isolation Kit (Miltenyi Biotec, Bergisch Gladbach, Germany) and cultured in AlyS505 NK-EX (CSTI, Japan) containing 10% autologous serum and 600 IU/mL interleukin-2 (Miltenyi Biotec).

All procedures were performed in compliance with relevant laws and institutional guidelines and have been approved by the Ethics Committee of the First Hospital of Jilin University according to Declaration of Helsinki principles (reference number: 2023-347, June 7, 2023). Informed consent was obtained for experimentation with human subjects.

### Cytotoxicity assay of NK cells

NK cell cytotoxicity was analyzed using the calcein-release assay as previously described (43). Briefly, NK cells (effector) were tested for cytotoxicity by incubating with K562 cells (target) that were labeled with 1 μg/ml calcein-AM (Dojindo Laboratories, Japan) for 30 min at 37 °C with occasional shaking. Effector cells and target cells were co-cultured at the indicated effector:target ratios of 10:1 and incubated at 37 °C for 4h. Then, 100μl of the supernatant was harvested and transferred to a new plate. Absorbance at 485 nm of excitation light wavelength and 528 nm of emission wavelength was determined using a Synergy™ HT multi-function MPP detector (BioTek, VT). The percent lysis was calculated according to the formula: specific lysis (%) = [(experimental release-spontaneous release)/(maximum release-spontaneous release)]×100.

### Measurement of INF-γ secretion

Primary NK cells and NK-92MI cells were treated with VPA (2mM for 24h) at a density of 2×10^6^ cells/ml. The supernatant was harvested and IFN-γ production was assayed using the Human IFN-γ ELISA Kit (Boster, Wuhan, China), according to the manufacturer’s instructions.

### RNA-Seq to identify differentially expressed lncRNAs in human NK cells

Total RNA was extracted from human cultured primary NK cells treated (2mM for 24h) and untreated with VPA by TRI REAGENT (Sigma, MO) according to the manufacturer’s guide (17). The indexed libraries were prepared using Illumina TruSeq RNA Library Preparation Kit v2. Paired-end sequencing in triplicate was performed by Shanghai Biotechnology Corporation (Shanghai, PRC) using a HiSeq4000 (Illumina). The RNA-Seq assay yielded 196 million raw reads and 183 million raw reads for VPA-treated and untreated human NK cells. After Seqtk filtering, a total of 159 million and 150 million clean reads for VPA-treated and untreated groups were mapped to the human genome (genome version: hg19, http://hgdownload.soe.ucsc.edu/goldenPath/hg19/bigZips) for mRNAs and lncRNAs using TopHat (RRID: SCR_013035) software. Gene counts were normalized to the values of fragments per kilobase of exon model per million mapped reads (FPKM). Cuffdiff (RRID: SCR_001647) was used to calculate the differentially expressed RNAs when the fold-change was > 2 and p < 0.05 with an unpaired two-sided t test.

### RNA extraction and quantitative PCR

Total RNA was extracted by TRI REAGENT (Sigma, MO), and M-MLV Reverse Transcriptase (Invitrogen, CA) was used to synthesize cDNA after removing genomic DNA contamination with DNase I (Sigma, MO) according to the manufacturer’s guide. Quantitative real-time PCR was performed in triplicate with the CFX96TM real-time system (BIO-RAD) using SYBR Prime Script TM RT-PCR Kit (Takara, CA). The mRNA expression levels of *NCR1* were calculated using threshold cycle (Ct) values standardized to β-ACTIN (housekeeping gene), applying the 2^(-ΔCt) method. The primers used for qPCR are listed in **Supplementary Table S1**.

### Knockdown of LINC02470 by shRNA lentiviruses

*LINC02470* was knocked down by two separate shRNA lentiviruses as previously described (44). The shRNA vector was constructed by cloning four shRNAs into two pGreenPuro vectors (#SI505A-1, SBI, CA). ShRNAs were designed online (https://portals.broadinstitute.org/gpp/public). For cloning, two pairs of shRNAs (5’-TTGAACTGActtcctgtcagaTCAGTTCAAACCAATATAAAGACGGttttttAAGGTCGGGCAGGAAGAG-3’; 5’-GGTTTGCAtctgacaggaagTGCAAACCGCAGGAAACTGACGACCggtgtttcgtcctttccacAAG-3’ and 5’-GAccacttGGATCCCCGTCTTTATATTGGTTTGAACTGActtcctgtcagaTCA-3’; 5’-gAatcgaaGAATTCaaaaaaGGTCGTCAGTTTCCTGCGGTTTGCAtctgacaggaagTGC-3’) (**Supplementary Table S1**) combined with loop were linked to the H1 and U6 promoter using PCR and were ligated into the EcoR1/BamH1 site in pGreenPuro vector. The copGFP reporter in the vector was used to track the lentiviral transfection in NK-92MI cells. Two random shRNAs (5′-GCAGCAACTGGACACGTGATCTTAA-3′ and 5′-TGAAATGTACTGCGCGTGGAGACTA-3) were cloned in the same vector as the assay control (shCT).

### LINC02470 overexpression

The full-length *LINC02470* lncRNA was amplified with PCR primers and ligated into the lenti-pCAG-Linc02470-pEF1-GFP/Puro vector constructed in our lab. After confirmed by DNA sequencing, the *LINC02470* plasmid DNA was packaged in 293T cells as previously described (44). The *LINC02470* lentiviruses were used to tranduce NK-92MI cells. For human primary NK cells, the overexpression assay was performed by transient transfection of *LINC02470*-overexpressing plasmids using Human Natural Killer Cell NucleofectorTM Kit (cat: VPA-1005; Lonza, Basel, Switzerland), following the protocol provided by the manufacturer.

### Lentivirus transfection

NK-92MI cells were seeded in six-well plates at 5×10^5^ cells/ml with 2 ml of medium containing 8μg/ml polybrene. Cells were infected with lentiviruses at a multiplicity of infection (MOI) of 10, centrifuged at 1800g for 45min at 37 °C. The medium was replaced every 2 days from 12-14h after infection.

### Knockdown of LINC02470 in primary human NK cells

A panel of siRNAs for *LINC02470* was designed and synthesized by GENCEFE Biotech (Wuxi, China). The primer sequences of siRNAs are listed in **Supplementary Table S1**. Primary human NK cells were transfected with 200nM siRNAs using Human Natural Killer Cell NucleofectorTM Kit (cat: VPA-1005; Lonza, Basel, Switzerland), following the protocol provided by the manufacturer.

### Cytoplasmic and nuclear fractions separation

A nucleocytoplasmic separation kit (Invitrogen, Carlsbad, CA, USA) was used to isolate RNA from nucleus and cytoplasm of NK-92MI cells. Then, PCR was performed to detect *LINC02470* in the nucleus and cytoplasm, respectively. β-Actin and U6 were used as controls. The primer information is listed in **Supplementary Table S1**.

### RNA fluorescence *in situ* hybridization

RNA fluorescence *in situ* hybridization was performed as previously reported (18). Briefly, NK-92MI cells were washed twice with RNase-free phosphate-buffered saline (PBS) after being fixed on glass slides by centrifugation. Then, cells were fixed with 4% paraformaldehyde on ice for 10 min, and freshly prepared 0.5% TritonX-100 was added. After dehydration and drying with ethanol, 10 µL of denatured digoxigenin-labeled RNA probe (50 ng total) was added dropwise onto the slide. Next, the slide was placed in a wet box and hybridized overnight at 42 °C. An antidigoxin-fluorescein antibody (Roche Diagnostics, Mannheim, Germany) was added after washing. The slides were incubated for 4 h and washed three times with PBS. DAPI (20 ng/mL; Beyotime, Shanghai, China) was added dropwise onto the slide and incubated for 10 min at 24 °C. The slides were imaged using an FV3000 confocal microscope (Olympus, Tokyo, Japan) after washing three times with PBS. The probe sequence information is listed in **Supplementary Table S1**.

### RNA reverse transcription-associated trap

RNA reverse transcription-associated trap (RAT) was performed as previously reported (45). NK-92MI cells were fixed with formaldehyde and were treated with a hypotonic solution (10 mM HEPES pH 7.9, 1.5 mM MgCl_2_, 10 mM KCl, 0.4% NP40) to lyse the cell membrane and isolate the nuclei. Reverse transcription was carried out using biotin-labeled dNTP Mix (Thermo Fisher Scientific) with specific primers complementary to *LINC02470* (**Supplementary Table S1**). The nucleus was lysed by ultrasonication, and biotinylated lncRNA-cDNA/chromatin DNA complexes were pulled down with streptavidin Dynabeads (Invitrogen). Protease K (Thermo Fisher Scientific) was added to degrade the protein and genomic DNA interacting with *LINC02470*.

### Flow cytometry analysis

NKp46 surface expression were analyzed with a FACSCalibur flow cytometer (BD Biosciences) using following mAbs: APC-anti-NKp46, PE-anti-CD56, PerCP-anti-CD3 and isotype-matched mAb (negative control for nonspecific binding) (all from BD Biosciences, CA).

### NKp46 antibody-blocking assay

To examine the role of NKp46 in NK cell killing in K-562 cells, we pre-incubated control NK-92MI cells and shLINC02470-treated NK-92MI cells with 10 μg/mL NKp46 blocking antibody (clone 9E2, BioLegend) at 37 °C for 1 hour. Cells were then co-cultured with K562 cells for cell killing. An isotype control IgG was employed as a control. NK cell cytotoxicity was analyzed using the calcein-release assay (43).

### Chromosome conformation capture

As previously reported (46), 1–5 million cells were crosslinked by 2% formaldehyde at room temperature for 10 min, and glycine was then used for quenching. Lysis buffer was used to lyse cells, followed by MboI (NEB, R0147) digestion at 37°C for at least 2 h. T4 DNA ligase (NEB, M0202) was used for ligation. Proteinase K was used to reverse the crosslinks, and DNA was then purified. The primers for 3C PCR were derived from various regions of *NCR1* (5’ enhancer and promoter). The 3C PCR products were inserted into a pJET Vector (Thermo, K1232) for sequencing. By checking the MboI ligation site, intrachromosomal interactions were confirmed. The 3C ligation products were quantified by qPCR.

### Methylation analysis by sodium bisulfite sequencing

Genomic DNA was isolated using the DNeasy Blood and Tissue kit (Qiagen) and modified with bisulfate using the EZ DNA Methylation Gold kit (ZYMO Research). DNA was amplified using specific primers for *NCR1* (**Supplementary Table S1**). PCR products were cloned by pJET PCR Cloning kit (Thermo Fisher Scientific, CA). Ten independent clones from each sample were sequenced to determine DNA methylation status.

### Chromatin immunoprecipitation assay

Chromatin immunoprecipitation (ChIP) assays were performed using the Pierce Agarose ChIP Kit (Thermo Fisher Scientific), according to the manufacturer’s instructions. Specific anti-trimethyl-H3K4, anti-trimethyl-H3K9, anti-acetyl-H3K27 antibody and anti-acetyl-H3K9 antibody (Cell Signaling Technology) were used to determine the promoter profile of *NCR1*. Normal rabbit IgG was used as a negative control. DNA was extracted and analyzed by qPCR with specific primers (**Supplementary Table S1**) targeting *NCR1* promoter. Enrichment was calculated using the following formula: ΔCt [normalized ChIP] = (Ct [ChIP] - (Ct [Input] -Log2 (Input Dilution Factor)), ΔΔCt [ChIP/NIS] = ΔCt [normalized ChIP] - ΔCt [IgG], Fold Enrichment = 2^ (-ΔΔCt [ChIP/NIS]).

### Statistical analysis

The experimental data were prepared and expressed as mean ± SD. Data were analyzed using SPSS software (version 16.0; SPSS, Inc., IL, USA, RRID: SCR_002865). Student’s *t*-test or one-way ANOVA (Bonferroni test) was performed to compare statistical differences among treatment groups. For every sample, two sets of duplicates were averaged for each of three biological replicates to obtain a final *n* of three for all statistical analyses. Results were considered statistically significant at P < 0.05. Error bars represent the standard deviation.

## Data Availability

The datasets presented in this study can be found in online repositories. The names of the repository/repositories and accession number(s) can be found below: https://www.ncbi.nlm.nih.gov/, GSE285271 https://www.ncbi.nlm.nih.gov/, GSE284721.

## References

[1] Rumgay H Ferlay J de Martel C Georges D Ibrahim AS Zheng R . Global, regional and national burden of primary liver cancer by subtype. Eur J Cancer. (2022) 161:108–18. doi: 10.1016/j.ejca.2021.11.023. PMID: 34942552

[2] Llovet JM Kelley RK Villanueva A Singal AG Pikarsky E Roayaie S . Hepatocellular carcinoma. Nat Rev Dis Primers. (2021) 7:6. doi: 10.1007/s11938-004-0002-8. PMID: 33479224 PMC13537433

[3] Ladd AD Duarte S Sahin I Zarrinpar A . Mechanisms of drug resistance in HCC. Hepatology. (2024) 79:926–40. doi: 10.1097/hep.0000000000000237. PMID: 36680397

[4] Cheng M Chen Y Xiao W Sun R Tian Z . NK cell-based immunotherapy for Malignant diseases. Cell Mol Immunol. (2013) 10:230–52. doi: 10.1038/cmi.2013.10. PMID: 23604045 PMC4076738

[5] Childs RW Carlsten M . Therapeutic approaches to enhance natural killer cell cytotoxicity against cancer: the force awakens. Nat Rev Drug Discov. (2015) 14:487–98. doi: 10.1038/nrd4506. PMID: 26000725

[6] Klingemann H . Challenges of cancer therapy with natural killer cells. Cytotherapy. (2015) 17:245–9. doi: 10.1016/j.jcyt.2014.09.007. PMID: 25533934

[7] Wu Y Kuang DM Pan WD Wan YL Lao XM Wang D . Monocyte/macrophage-elicited natural killer cell dysfunction in hepatocellular carcinoma is mediated by CD48/2B4 interactions. Hepatology. (2013) 57:1107–16. doi: 10.1002/hep.26192. PMID: 23225218

[8] Fathy A Eldin MM Metwally L Eida M Abdel-Rehim M . Diminished absolute counts of CD56dim and CD56bright natural killer cells in peripheral blood from Egyptian patients with hepatocellular carcinoma. Egypt J Immunol. (2009) 16:17–25. 22059350

[9] Cai L Zhang Z Zhou L Wang H Fu J Zhang S . Functional impairment in circulating and intrahepatic NK cells and relative mechanism in hepatocellular carcinoma patients. Clin Immunol. (2008) 129:428–37. doi: 10.1016/j.clim.2008.08.012. PMID: 18824414

[10] Hoechst B Voigtlaender T Ormandy L Gamrekelashvili J Zhao F Wedemeyer H . Myeloid derived suppressor cells inhibit natural killer cells in patients with hepatocellular carcinoma via the NKp30 receptor. Hepatology. (2009) 50:799–807. doi: 10.1002/hep.23054. PMID: 19551844 PMC6357774

[11] Jinushi M Takehara T Tatsumi T Hiramatsu N Sakamori R Yamaguchi S . Impairment of natural killer cell and dendritic cell functions by the soluble form of MHC class I-related chain A in advanced human hepatocellular carcinomas. J Hepatol. (2005) 43:1013–20. doi: 10.1016/j.jhep.2005.05.026. PMID: 16168521

[12] Chew V Chen J Lee D Loh E Lee J Lim KH . Chemokine-driven lymphocyte infiltration: an early intratumoral event determining long-term survival in resectable hepatocellular carcinoma. Gut. (2012) 61:427–38. doi: 10.1136/gutjnl-2011-300509. PMID: 21930732 PMC3273680

[13] Jewett A Tseng HC . Tumor induced inactivation of natural killer cell cytotoxic function; implication in growth, expansion and differentiation of cancer stem cells. J Cancer. (2011) 2:443–57. doi: 10.7150/jca.2.443. PMID: 21850212 PMC3157021

[14] Wright PW Huehn A Cichocki F Li H Sharma N Dang H . Identification of a KIR antisense lncRNA expressed by progenitor cells. Genes Immun. (2013) 14:427–33. doi: 10.1038/gene.2013.36. PMID: 23863987 PMC3808466

[15] Zhang R Ni F Fu B Wu Y Sun R Tian Z . A long noncoding RNA positively regulates CD56 in human natural killer cells. Oncotarget. (2016) 7:72546–58. doi: 10.18632/oncotarget.12466. PMID: 27713137 PMC5341928

[16] Niu C Li M Chen Y Zhang X Zhu S Zhou X . LncRNA NCAL1 potentiates natural killer cell cytotoxicity through the Gab2-PI3K-AKT pathway. Front Immunol. (2022) 13:970195. doi: 10.3389/fimmu.2022.970195. PMID: 36248894 PMC9554105

[17] Shi X Li M Cui M Niu C Xu J Zhou L . Epigenetic suppression of the antitumor cytotoxicity of NK cells by histone deacetylase inhibitor valproic acid. Am J Cancer Res. (2016) 6:600–14. PMC4851840 27152238

[18] Du Z Wen X Wang Y Jia L Zhang S Liu Y . Chromatin lncRNA Platr10 controls stem cell pluripotency by coordinating an intrachromosomal regulatory network. Genome Biol. (2021) 22:233. doi: 10.1186/s13059-021-02444-6. PMID: 34412677 PMC8375132

[19] Zhu Y Yan Z Fu C Wen X Jia L Zhou L . LncRNA Osilr9 coordinates promoter DNA demethylation and the intrachromosomal loop structure required for maintaining stem cell pluripotency. Mol Ther. (2023) 31:1–16. doi: 10.1016/j.ymthe.2022.12.010. PMID: 36523163 PMC10278046

[20] Song C Zhang G Mu X Feng C Zhang Q Song S . eRNAbase: a comprehensive database for decoding the regulatory eRNAs in human and mouse. Nucleic Acids Res. (2024) 52:D81–91. doi: 10.1093/nar/gkad925. PMID: 37889077 PMC10767853

[21] Guo CJ Xu G Chen LL . Mechanisms of long noncoding RNA nuclear retention. Trends Biochem Sci. (2020) 45:947–60. doi: 10.1016/j.tibs.2020.07.001. PMID: 32800670

[22] Mandelboim O Porgador A . NKp46. Int J Biochem Cell Biol. (2001) 33:1147–50. doi: 10.1016/s1357-2725(01)00078-4. PMID: 11606250

[23] Barrow AD Martin CJ Colonna M . The natural cytotoxicity receptors in health and disease. Front Immunol. (2019) 10:909. doi: 10.3389/fimmu.2019.00909. PMID: 31134055 PMC6514059

[24] Santara SS Lee D-J Crespo Â Hu JJ Walker C Ma X . The NK cell receptor NKp46 recognizes ecto-calreticulin on ER-stressed cells. Nature. (2023) 616:348–56. doi: 10.1101/2021.10.31.466654. PMID: 37020026 PMC10165876

[25] Cuartero S Weiss FD Dharmalingam G Guo Y Ing-Simmons E Masella S . Control of inducible gene expression links cohesin to hematopoietic progenitor self-renewal and differentiation. Nat Immunol. (2018) 19:932–41. doi: 10.1038/s41590-018-0184-1. PMID: 30127433 PMC6195188

[26] Abe S Funato T Takahashi S Yokoyama H Yamamoto J Tomiya Y . Increased expression of insulin-like growth factor i is associated with Ara-C resistance in leukemia. Tohoku J Exp Med. (2006) 209:217–28. doi: 10.1620/tjem.209.217. PMID: 16778368

[27] Calo E Wysocka J . Modification of enhancer chromatin: what, how, and why? Mol Cell. (2013) 49:825–37. doi: 10.1016/j.molcel.2013.01.038. PMID: 23473601 PMC3857148

[28] Lin YC Benner C Mansson R Heinz S Miyazaki K Miyazaki M . Global changes in the nuclear positioning of genes and intra- and interdomain genomic interactions that orchestrate B cell fate. Nat Immunol. (2012) 13:1196–208. doi: 10.1038/ni.2432. PMID: 23064439 PMC3501570

[29] Kagey MH Newman JJ Bilodeau S Zhan Y Orlando DA van Berkum NL . Mediator and cohesin connect gene expression and chromatin architecture. Nature. (2010) 467:430–5. doi: 10.1038/nature09380. PMID: 20720539 PMC2953795

[30] Mousavi K Zare H Dell'orso S Grontved L Gutierrez-Cruz G Derfoul A . eRNAs promote transcription by establishing chromatin accessibility at defined genomic loci. Mol Cell. (2013) 51:606–17. doi: 10.1016/j.molcel.2013.07.022. PMID: 23993744 PMC3786356

[31] Wang C Jia L Wang Y Du Z Zhou L Wen X . Genome-wide interaction target profiling reveals a novel Peblr20-eRNA activation pathway to control stem cell pluripotency. Theranostics. (2020) 10:353–70. doi: 10.7150/thno.39093. PMID: 31903125 PMC6929617

[32] Igolkina AA Zinkevich A Karandasheva KO Popov AA Selifanova MV Nikolaeva D . H3K4me3, H3K9ac, H3K27ac, H3K27me3 and H3K9me3 histone tags suggest distinct regulatory evolution of open and condensed chromatin landmarks. Cells. (2019) 8. doi: 10.3390/cells8091034. PMID: 31491936 PMC6770625

[33] Becker JS Nicetto D Zaret KS . H3K9me3-dependent heterochromatin: barrier to cell fate changes. Trends Genet. (2016) 32:29–41. doi: 10.1016/j.tig.2015.11.001. PMID: 26675384 PMC4698194

[34] Ruthenburg AJ Allis CD Wysocka J . Methylation of lysine 4 on histone H3: intricacy of writing and reading a single epigenetic mark. Mol Cell. (2007) 25:15–30. doi: 10.1016/j.molcel.2006.12.014. PMID: 17218268

[35] Kulis M Esteller M . DNA methylation and cancer. Adv Genet. (2010) 70:27–56. doi: 10.1016/b978-0-12-380866-0.60002-2. PMID: 20920744

[36] Baytak E Gong Q Akman B Yuan H Chan WC Kucuk C . Whole transcriptome analysis reveals dysregulated oncogenic lncRNAs in natural killer/T-cell lymphoma and establishes MIR155HG as a target of PRDM1. Tumor Biol. (2017) 39:1010428317701648. doi: 10.1177/1010428317701648. PMID: 28468592

[37] Liu Q Ran R Wu Z Li X Zeng Q Xia R . Long non-coding RNA X-inactive specific transcript mediates cell proliferation and intrusion by modulating the miR-497/bcl-w axis in extranodal natural killer/T-cell lymphoma. Front Cell Dev Biol. (2020) 8:599070. doi: 10.3389/fcell.2020.599070. PMID: 33364236 PMC7753184

[38] Guo F Yuan Y Chen Z Gao F Li X Wang H . Downregulation of the long non-coding RNA MALAT1 in tenofovir-treated pregnant women with hepatitis B virus infection promotes immune recovery of natural killer cells via the has-miR-155-5p/HIF-1alpha axis. Int Immunopharmacol. (2022) 107:108701. doi: 10.2139/ssrn.3993202. PMID: 35306280

[39] Li S Zhu A Ren K Li S Chen L . IFNbeta-induced exosomal linc-EPHA6–1 promotes cytotoxicity of NK cells by acting as a ceRNA for hsa-miR-4485-5p to up-regulate NKp46 expression. Life Sci. (2020) 257:118064. doi: 10.1016/j.lfs.2020.118064. PMID: 32652136

[40] Huang Y Luo Y Ou W Wang Y Dong D Peng X . Exosomal lncRNA SNHG10 derived from colorectal cancer cells suppresses natural killer cell cytotoxicity by upregulating INHBC. Cancer Cell Int. (2021) 21:528. doi: 10.1186/s12935-021-02221-2. PMID: 34641864 PMC8507338

[41] Wei MF Gu ZS Zheng LL Zhao MX Wang XJ . Long non-coding RNA GAS5 promotes natural killer cell cytotoxicity against gastric cancer by regulating miR-18a. Neoplasma. (2020) 67:1085–93. doi: 10.4149/neo_2020_191014n1034. PMID: 32538667

[42] Li T Li X Guo Y Zheng G Yu T Zeng W . Distinct mRNA and long non-coding RNA expression profiles of decidual natural killer cells in patients with early missed abortion. FASEB J. (2020) 34:14264–86. doi: 10.1096/fj.202000621r. PMID: 32915478

[43] Niu C Chen Y Li M Zhu S Zhou L Xu D . Non-coated rituximab induces highly cytotoxic natural killer cells from peripheral blood mononuclear cells via autologous B cells. Front Immunol. (2021) 12:658562. doi: 10.3389/fimmu.2021.658562. PMID: 34113342 PMC8185348

[44] Sun J Li W Sun Y Yu D Wen X Wang H . A novel antisense long noncoding RNA within the IGF1R gene locus is imprinted in hematopoietic Malignancies. Nucleic Acids Res. (2014) 42:9588–601. doi: 10.1093/nar/gku549. PMID: 25092925 PMC4150754

[45] Zhao Y Zhou L Li H Sun T Wen X Li X . Nuclear-Encoded lncRNA MALAT1 Epigenetically Controls Metabolic Reprogramming in HCC Cells through the Mitophagy Pathway. Mol Ther Nucleic Acids. (2021) 23:264–76. doi: 10.1016/j.omtn.2020.09.040. PMID: 33425485 PMC7773746

[46] Jia L Wang Y Wang C Du Z Zhang S Wen X . Oplr16 serves as a novel chromatin factor to control stem cell fate by modulating pluripotency-specific chromosomal looping and TET2-mediated DNA demethylation. Nucleic Acids Res. (2020) 48:3935–48. doi: 10.1093/nar/gkaa097. PMID: 32055844 PMC7144914

