# Supplementary material for: LINC02470 impairs natural killer cell cytotoxicity by epigenetically targeting the natural cytotoxicity triggering receptor 1
Source: Front Immunol. 2026 Apr 16;17:1627089. doi: 10.3389/fimmu.2026.1627089 (PMC13128666; doi:10.3389/fimmu.2026.1627089)
Supplement: Supplementary file 1 [file SupplementaryFile1.pdf]

LINC02470 impairs natural killer cell cytotoxicity by epigenetically targeting the Natural Cytotoxicity Triggering Receptor 1 (_NCR1_)

Xiumin Shi[1], Lei Zhou[2], Min Li[2], Xue Wen[2], Yongchong Chen[2], Chao Niu[2], Qiliang Yin[3], Haofan Jin[2], Andrew R. Hoffman[4], Jiuwei Cui[2*], Sujun Gao[1*], Ji-Fan Hu[2,4*]

[1] Department of Hematology, First Hospital, Jilin University, Changchun, Jilin 130021, P.R. China

[2] Cancer Center, First Hospital, Jilin University, Changchun, Jilin 130021, P.R. China

[3] Department of Cadre Ward, First Hospital, Jilin University, Changchun, Jilin 130021, P.R. China

[4] Stanford University Medical School, VA Palo Alto Health Care System, Palo Alto, CA 94304, USA

Supplemental Figures

Figure S1. Top 30 of GO enrichment in RNA-seq.

Gene ontology (GO) analysis revealed that genes are involved in many signal pathways. For example, extracellular region, membrane, protein complex, response to stress and immune system process.

Figure S2. KEGG classification of RNA-seq.

KEGG classification of RNA-seq. Kyoto Encyclopedia of Genes and Genomes (KEGG) analysis showed that immune system and immune diseases were the main areas.

Figure S3. Top 30 of pathway enrichment in RNA-seq.

The result showed that there were many signal pathways that are related to immune system.

Figure S4. *LINC02470* is located in a NK function locus.

A. *LINC02470* was located in a unique NK cell functional domain on chromosome 12, containing the C-type lectin domain family member cluster and the killer cell lectin-like receptor family members.

B. The IGV plot of *LINC02470* from RNA-seq data of NK cells treated with VPA and PBS. *LINC02470* (Gene ID NONHSAT026854) is located at chromosome 12: (chr12: 9,936,579-9,943,495). Both VPA and PBS treated

NK cells RNA-seqBam data were analyzed by IGV, and the Sashimi plot was acquired for *LINC02470*.

C. The location and exons of *LINC02470*.

Figure S5. Reduced function of HCC NK cells and NK-92MI cells by VPA.

A. VPA-induced cytotoxicity suppression of NK cells from hepatocellular carcinoma (HCC) patients (n=10) and NK-92MI cells. NK cells were treated with histone deacetylase inhibitor valproic acid VPA (2 μm for 24h) and were evaluated for NK cell cytotoxicity. *** $p < 0.001$.

B. Decreased IFN-γ secretion in HCC NK cells (n=10) and NK-92MI cells after treatment with histone deacetylase inhibitor VPA. *** $p < 0.001$.

Figure S6. GFP expression in NK-92MI cells after shRNA lentiviruses transfection.

shCT: NK-92MI transfected with the random shRNA vector control; shLINC02470: NK-92MI transfected with shLINC02470.

Figure S7. Top 20 of Gene Ontology enrichment analysis of the *LINC02470* RAT-seq data. GO enrichment was analyzed with Cytoscape software.

Figure S8. *LINC02470* does not affect DNA methylation in the *NCR1* promoter.

A. CpG islands in the *NCR1* promoter. 5'-Enh: 5'-enhancer; 3'-Enh: 3'-enhancer; p*NCR1*: *NCR1* promoter; E1-E7: exons.

B. *LINC02470* has no effect on DNA methylation. Cells were collected for measurement of DNA methylation by sodium bisulfite sequencing. Solid dot: methylated CpG islands; open dot: unmethylated CpG islands. Numbers under CpG sites: the percentage of methylated CpGs over total CpGs in the sequencing. Each line represents the sequence for one clone. A total of 10 clones were sequenced for each group.

Figure S9. *LINC02470* does not affect H3K27ac and H3K9ac in the *NCR1* promoter.

Enrichments of *NCR1* DNA sequences for H3K27ac and H3K9ac occupancy were measured using ChIP-qPCR. Data were obtained from three independent experiments.

Figure S10. Ecto-calreticulin expression in K562 cells and healthy BM cells.

The expression of ecto-calreticulin (ecto-CRT) in K562 cells and bone marrow cells from healthy donors (n=4) were detected by RT-qPCR. Ecto-CRT relative expression was calculated by applying the $2^{(-\Delta Ct)}$ method. ** $p < 0.01$.

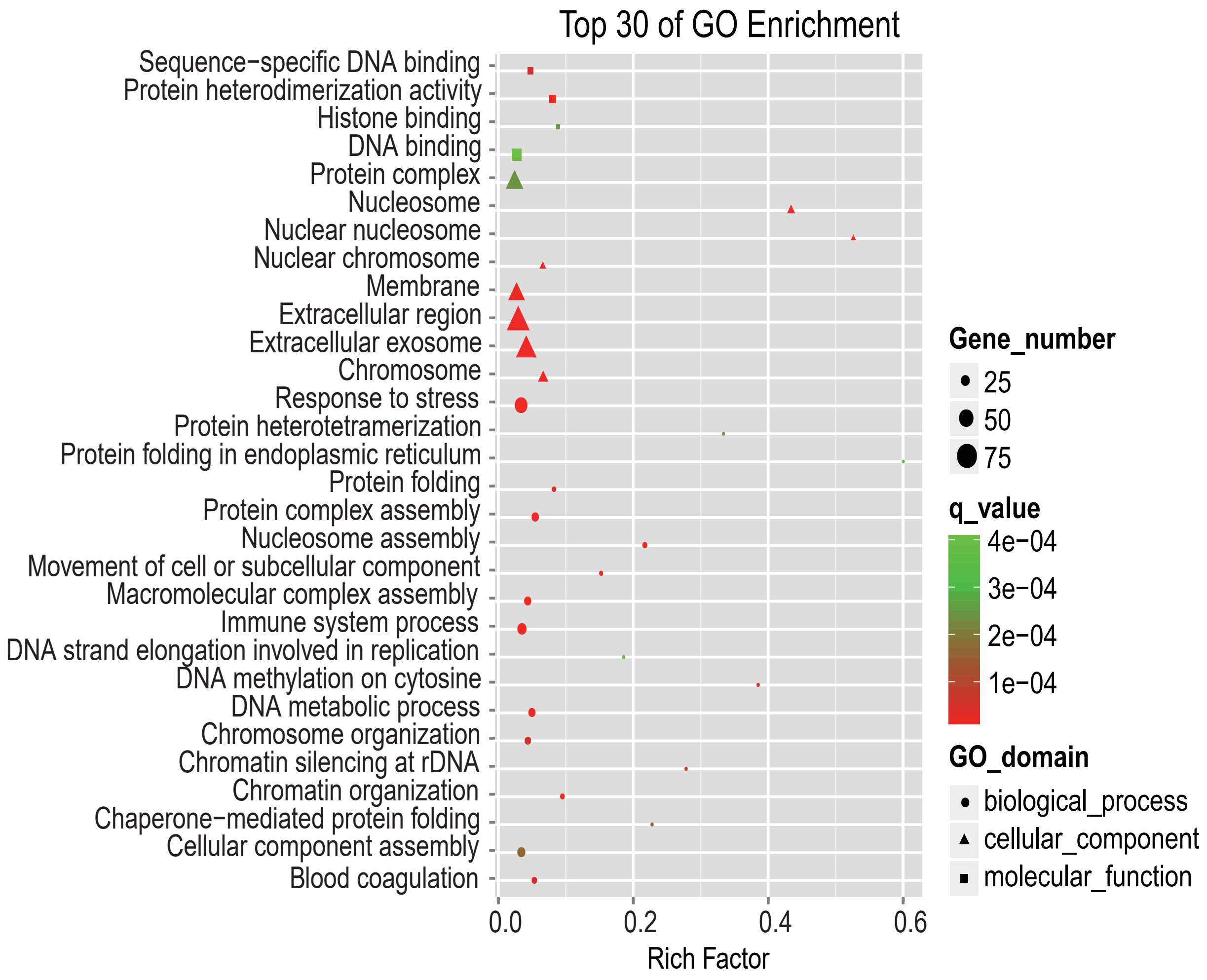

Figure S1. Top 30 of GO enrichment in RNA-seq

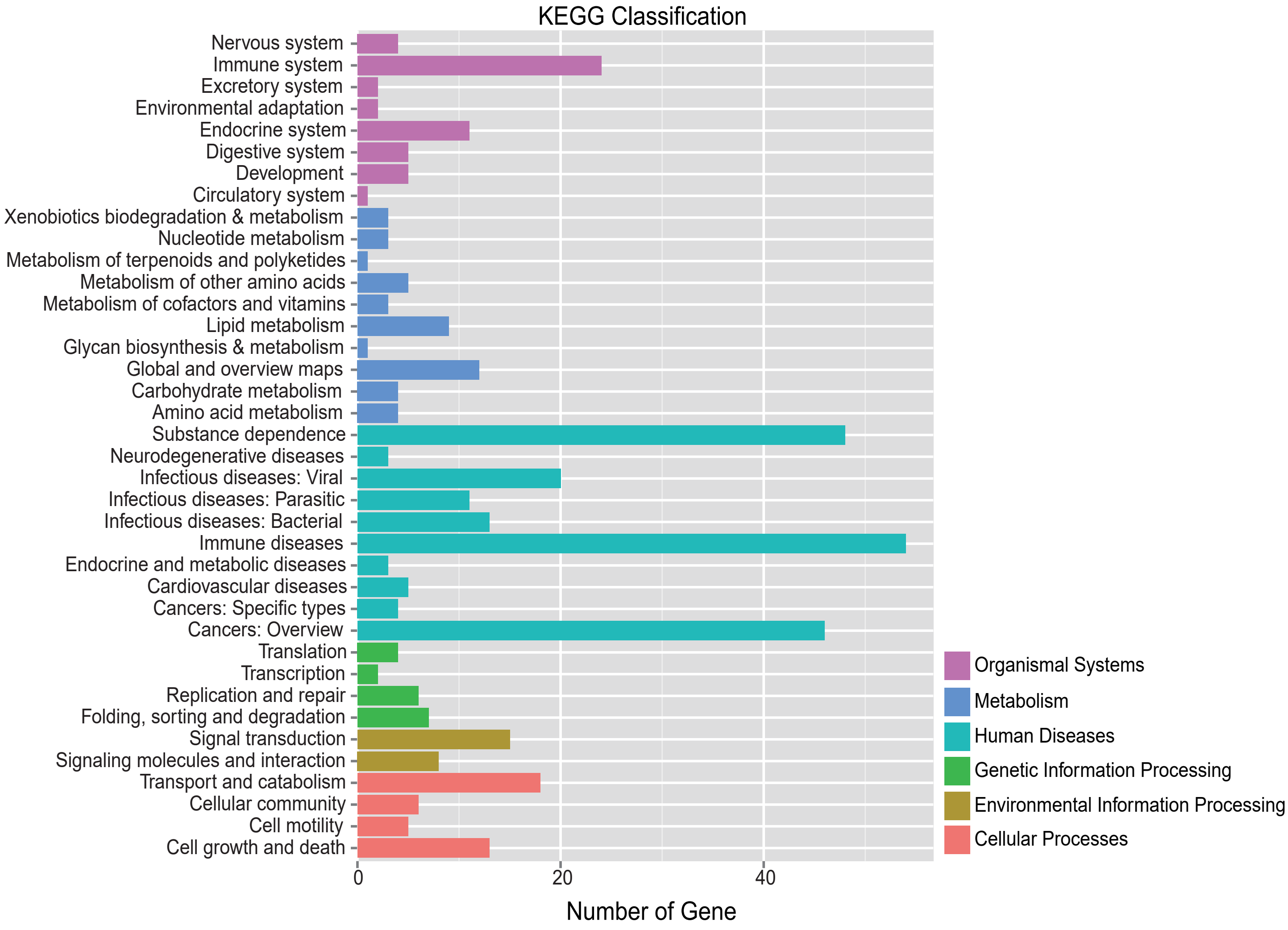

Figure S2. KEGG classification of RNA-seq

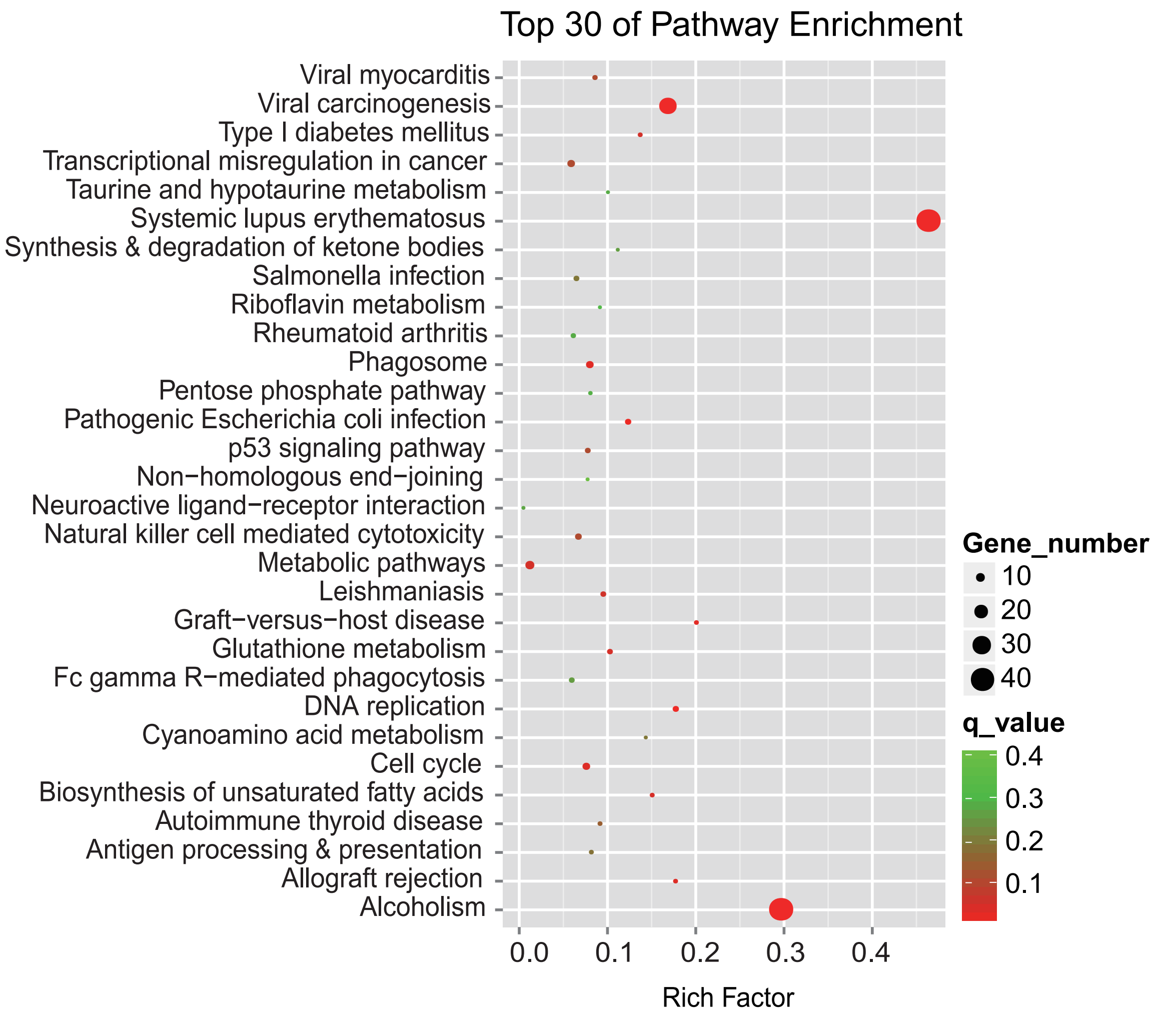

Figure S3. Top 30 of pathway enrichment in RNA-seq

A. *LINC02470* is located in a NK function locus

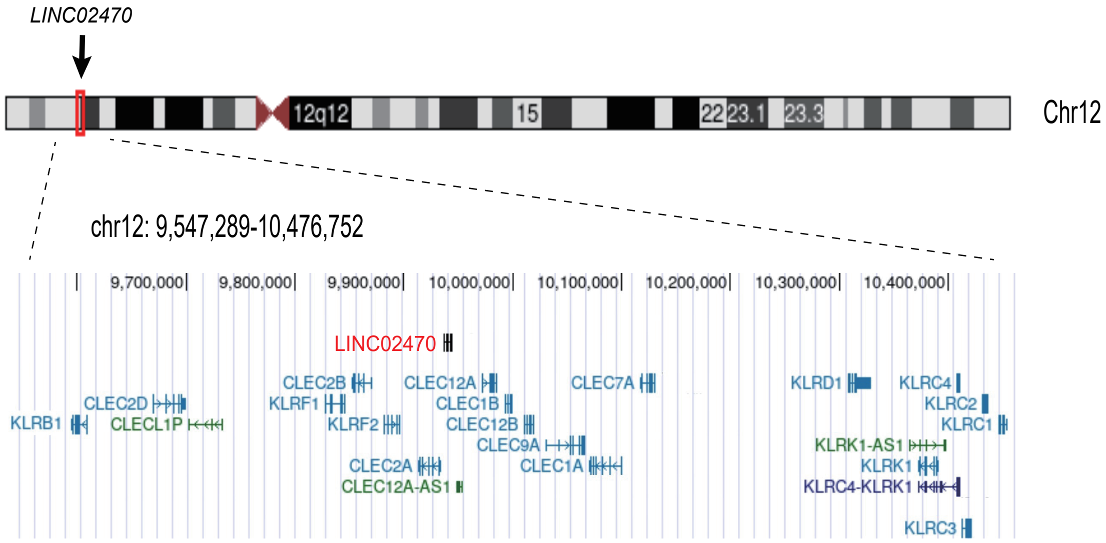

B. RNA-Seq IGV data

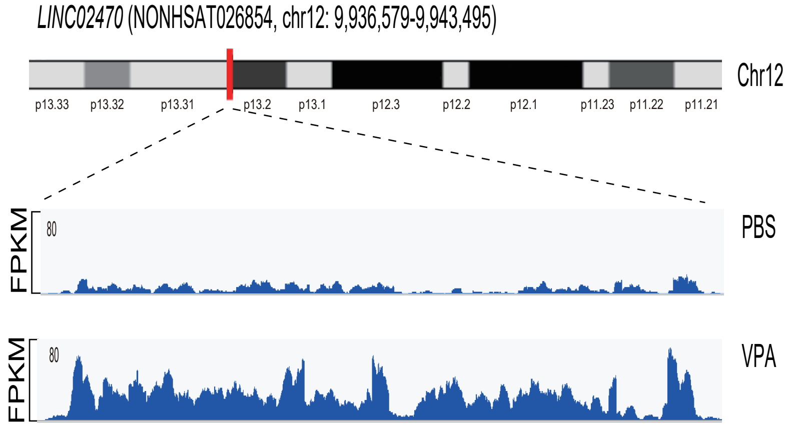

C. Location of *LINC02470* exons

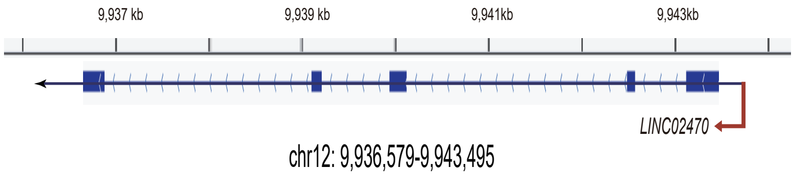

Figure S4. *LINC02470* is located in a NK function locus

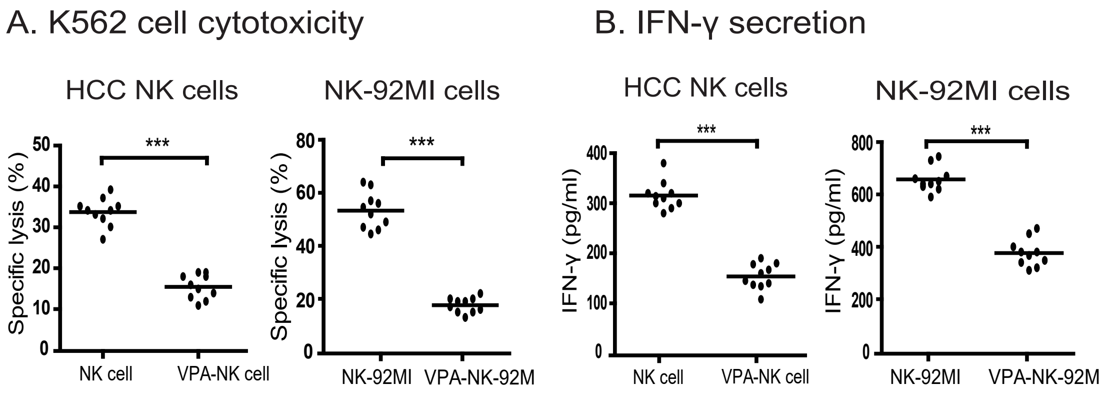

Figure S5. Reduced function of HCC NK cells and NK-92MI cells by VPA.

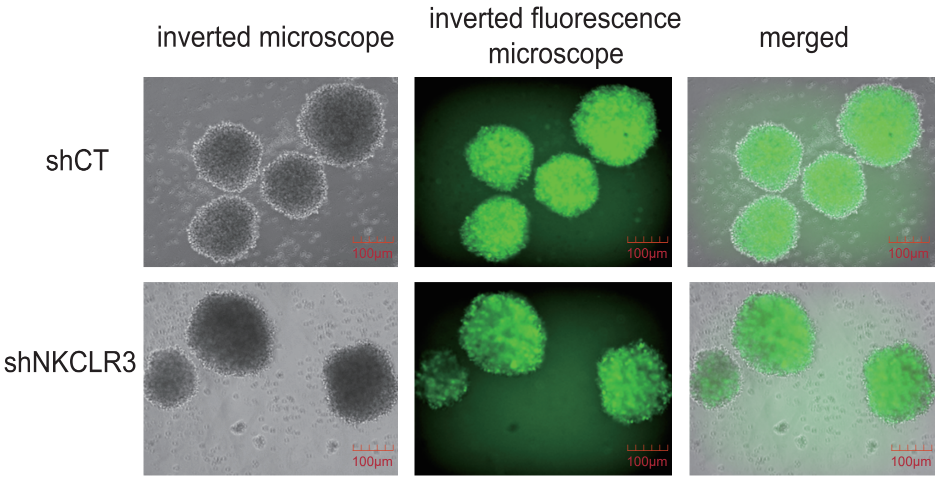

Figure S6. GFP expression in NK-92MI cells after shRNA lentiviruses transfection.

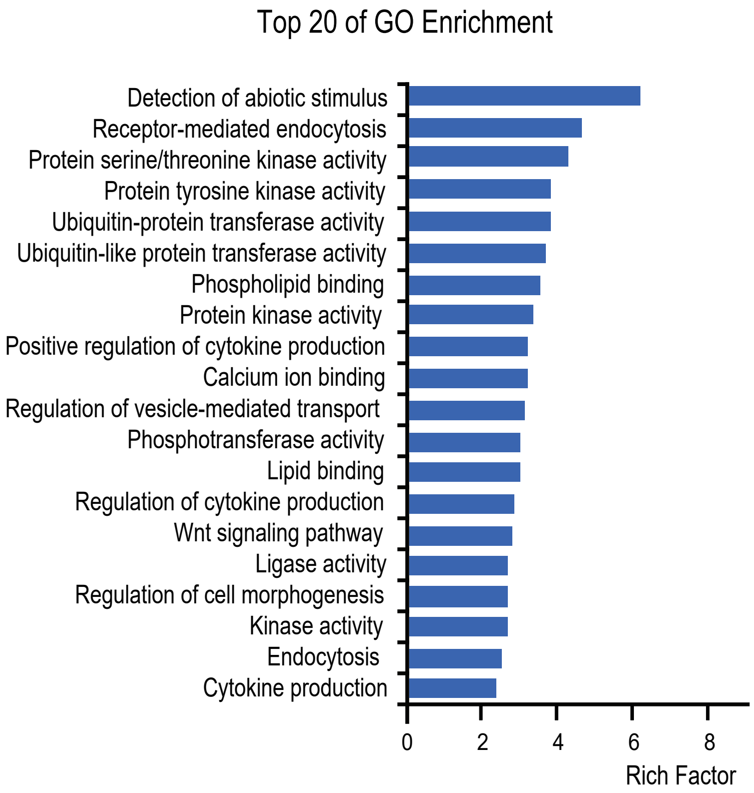

Figure S7. Top 20 of GO enrichment in *NKCLR3* RAT-seq.

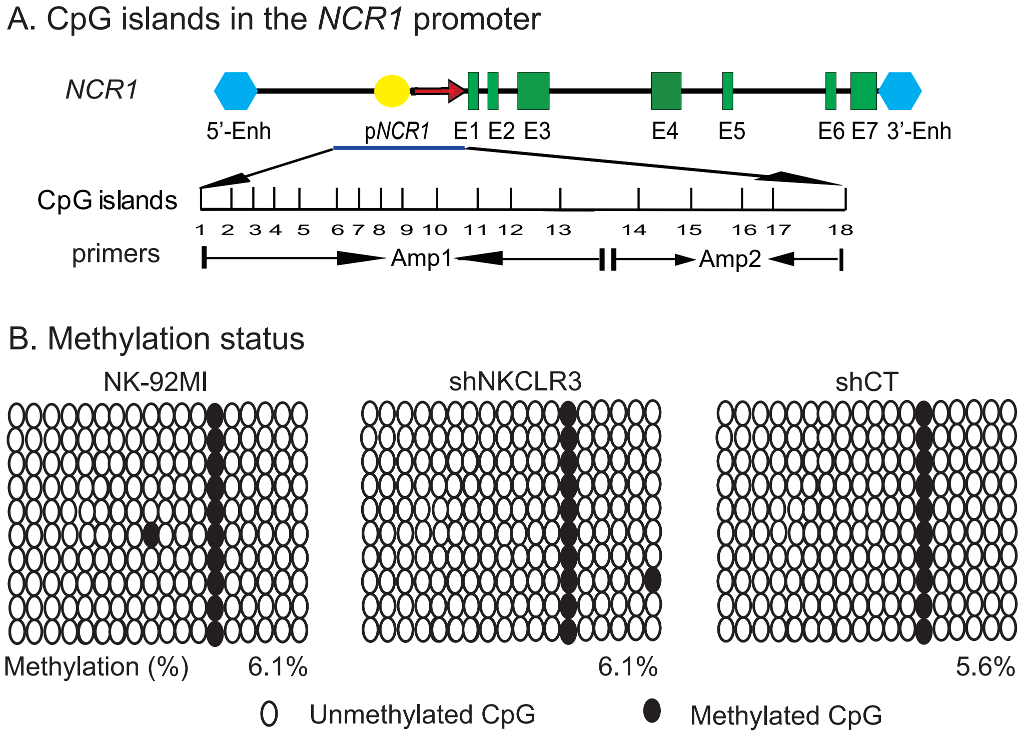

Figure S8. *NKCLR3* has no influence on DNA methylation ststus in the *NCR1* promoter.

ChIP-qPCR for H3K27ac and H3K9ac

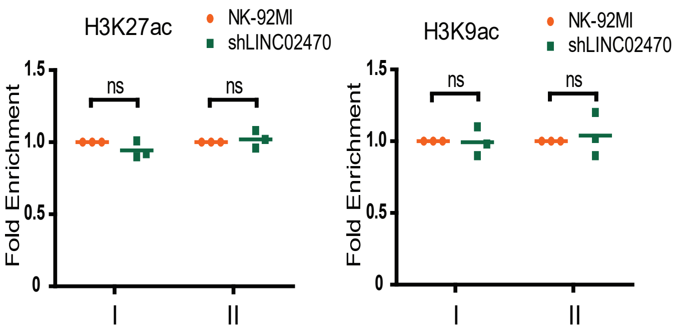

Figure S9. *LINC02470* has no effect on H3K27ac and H3K9ac level.

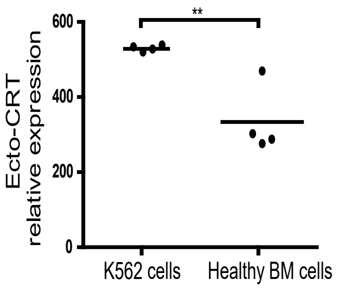

Figure S10. Ecto-calreticulin expression in K562 cells and healthy donors

Table S1. Oligonucleotide primers and shRNA used for *LINC02470*

Experiments	Target	Oligo sequence (5′-3′ direction)
RT-PCR	*LINC02470*	Sense CCTGCCTCGTACAAACGTCA
		Antisense ATTTGGTGAGTGCAAACCGC
	β- Actin	Sense AGATCAAGATCATTGCTCCTCCTGA
		Antisense ATACTCCTGCTTGCTGATCCACATC
	ecto-calreticulin	
		Sense AGCCTGCCGTCTACTTCAAG
		Antisense CGTAGAACTTGCCGGAACTG
sh*LINC02470*	I	Sense TTGAACTGACTTCCTGTCAGATCAGTTCAAACCAATATAAAGACGGTTTTTAAGGTCGGGCAGGAAGAG(JH5869)
		Antisense GGTTTGCATCTGACAGGAAGTGCAAACCGCAGGAAACTGACGACCGGTGTTTCGTCCTTTCCACAAG(JH5871)
	II	Sense GACCACTTGGATCCCCGTCTTTATATTGGTTTGAACTGACTTCCTGTCAGATCA(JH5870)
		Antisense GAATCGAAGAATTCAAAAAAGGTCGTCAGTTTCCTGCGGTTTGCATCTGACAGGAAGTGC(JH5872)
shCT	I	Antisense GCAGCAACTGGACACGTGATCTTAA
	II	Antisense TGAAATGTACTGCGCGTGGAGACTA
LINC02470-overexpression		Sense TTTTGGCAAAACCGGTGTGCATGCCCGGCTGCCGTTGC (SJ4564)
		Antisense GAAGTTATGATGCTAGCTAAAATATTAAGAGGTGCAGAGACT(SJ4565)
siRNAs	*LINC02470*-human-193	Sense GGUUUGCACUCACCAAAUATT
		Antisense UAUUUGGUGAGUGCAAACCTT
	LINC02470-human-513	Sense GGCUGGAACUGAAUCAAUATT
		Antisense UAUUGAUUCAGUUCCAGCCTT
	LINC02470-human-956	Sense GUCUCUGCACCUCUUAAUATT
		Antisense UAUUAAGAGGUGCAGAGACTT

LINC02470 localization assay	*LINC02470*	Sense CCTGCCTCGTACAAACGTCA
		Antisense ATTTGGTGAGTGCAAACCGC
	U6	Sense GTGCTCGCTTCGGCAGCACATATAC
		Antisense ATATGGAACGCTTCACGAATTTGCG
	β- Actin	Sense AGATCAAGATCATTGCTCCTCCTGA
		Antisense ATACTCCTGCTTGCTGATCCACATC
RNA FISH	RNA probe	Sense GATCTGCGTAATACGACTCACTATAGGGAGACCTGCCTCGTACAAACGTCA
		Antisense ATTTGGTGAGTGCAAACCGC
RAT	*LINC02470* specific primers	Antisense TGGTGCGGCAGCAAGACAGT
		Antisense CCCCCAAAGAAGAAAAGCAATGC
		Antisense CCAGCCTTGTGGACACATAGTC
	Random primer	Antisense ATGGACTGATGATCTTATGC
		Antisense TACATAGTAGATCAGATACT
RAT qPCR	5’-CT	Sense GGAGGGGAGATGTCAGATGG
		Antisense TACACATCGGTCCCTCCCTA
	5’-Enhancer (5’-Enh)	Sense TTTTAAAGGAGGCTGGGCGT
		Antisense ATTAGAGGCACCCACAACCA
	Promoter-1 (Prot-1)	Sense CACAACTTCCCACAGTCAGC
		Antisense AGAACGTTCACAACTCCCCT
	Promoter-2 (Prot-2)	Sense GTATCTATCTCCCTGGCCCG
		Antisense TTGAAGGAAGGACTCACGCT
	Exon 4 (E4)	Sense TGGACCCGAAGTGATCTCG
		Antisense TCCTTGAGCAGTAAGAACATGC
	Exon 7 (E7)	Sense TGGCCTTTCTAGTCCTGGTG
		Antisense CATGGCCACTGTGTCTCATG
	3’-Enhancer (3’-Enh)	Sense TGCAACCTCTAGCTCTCCAT

		Antisense AAGGCGGGGAGATCACTTG
	3'-CT	Sense AGACCCAAAGTACAGCGAGC
		Antisense ATGTCTTTACCCCTGCTCCC
3C	5'-CT	Antisense GACCCCACTGAAAATCGGACT(SJ3672)
		Antisense CAACTCACCTGGCAGCCACT(SJ3673)
	5'-Enhancer-1 (5'-Enh-1)	Antisense ACACAATTTATTTAATTGTCATGTC(SJ3674)
		Antisense GTCTCTCTTATTTGGAGATGGAATC(SJ3675)
	5'-Enhancer-2 (5'-Enh-2)	Antisense TGCATATGATGCCCTAAACCTCTA(SJ3676)
		Antisense TCCCAAAACCAAGGACATTCTG(SJ3677)
	Promoter-1 (Prot-1)	Sense AGGTGCACCGGCCCCAGTCA(SJ3678)
		Sense GACTGAGGCTCGAACAGAGAGT(SJ3679)
	Promoter-2 (Prot-2)	Sense CTAGACAGGATAACCTCAAGTCGT(SJ3680)
		Sense CATGTCTCAATTGAATAACTGCCT(SJ3681)
	Promoter-3 (Prot-3)	Sense CACTGCTCAGCACTAGGCCG(SJ3682)
		Sense CGGCAGAATCTGAGCGATGTC(SJ3683)
	3'-CT	Sense CACACCCAGCTACTTGTGCT(SJ3684)
		Sense TACTTGTGCTTTTTGACCAACATC(SJ3685)
BSP	I	Sense TAGATTATAGATGTGTTAGAGGGAT(SJ3668)
		Antisense ATTCCCCTTCCACGCCAAAACTCAC(SJ3669)
	II	Sense TGTTGTTAGGGAAATTATGGGGTTG(SJ3670)
		Antisense ATTACCTATTACCACCAAATCCAAC(SJ3671)
ChIP qPCR	I	Sense CACAACTTCCCACAGTCAGC
		Antisense AGAACGTTCACAACTCCCCT
	II	Sense GTATCTATCTCCCTGGCCCG
		Antisense TTGAAGGAAGGACTCACGCT
5'eRNA	I	Sense TTTTAAAGGAGGCTGGGCGT
		Antisense ATTAGAGGCACCCACAACCA
	II	Sense TGGTTGTGGGTGCCTCTAAT
		Antisense TTCACCACTTGTTTTACCAGTG
3'eRNA	I	Sense TGCAACCTCTAGCTCTCCAT
		Antisense AAGGCGGGGAGATCACTTG
	II	Sense AAGTGATCTCCCCGCCTTG
		Antisense ATTTCCATGGCCATCCTGTT
